# Economic evaluation of six immune checkpoint inhibitor-based first-line therapies for advanced squamous non-small-cell lung cancer: a cost-effectiveness analysis based on network meta-analysis

**DOI:** 10.3389/fphar.2026.1841871

**Published:** 2026-10-13

**Authors:** Zijian Qiu, Guodong Wang, Ying Lou, Feng Xuan

**Affiliations:** 1 Department of Radiation Oncology, The Quzhou Affiliated Hospital of Wenzhou Medical University, Quzhou People’s Hospital, Quzhou, China; 2 Department of Radiation Oncology, Zhuji Affiliated Hospital of Wenzhou Medical University, Shaoxing, China; 3 Department of Medical Oncology, Zhuji Affiliated Hospital of Wenzhou Medical University, Shaoxing, China

**Keywords:** cost-effectiveness analysis, first-line therapy, immune checkpoint inhibitors, network meta-analysis, non-small cell lung cancer, partitioned survival model

## Abstract

**Background:**

Advanced squamous non-small-cell lung cancer (sq-NSCLC) imposes a substantial clinical and economic burden. Immune checkpoint inhibitors (sugemalimab, tislelizumab, penpulimab, serplulimab, camrelizumab, and pembrolizumab) combined with chemotherapy improve survival, but their comparative cost-effectiveness remains uncertain.

**Methods:**

A three-state partitioned survival model with 3-week cycles and a lifetime horizon was developed from the Chinese healthcare-system perspective. Clinical efficacy was derived from six phase 3 trials and synthesized using network meta-analysis. Costs, life-years (LYs), quality-adjusted life-years (QALYs), and incremental cost-effectiveness ratios (ICERs) for each ICI-based regimen versus chemotherapy were estimated. The willingness‐to‐pay (WTP) threshold was set at $27,906/QALY. Deterministic, probabilistic, scenario, and full incremental analyses were performed.

**Results:**

In the base-case analysis, chemotherapy alone yielded 0.71 QALYs at $12,227.47. All ICI-based regimens increased QALYs to between 1.24 and 2.07. Pembrolizumab plus chemotherapy provided the greatest health benefit, yielding 2.07 QALYs, but had an ICER of $70,913.04 per QALY versus chemotherapy alone. Penpulimab plus chemotherapy had the lowest ICER versus chemotherapy alone ($13,064.23 per QALY), followed by camrelizumab ($13,361.50/QALY), and tislelizumab plus chemotherapy ($14,022.95/QALY), all below the WTP threshold. Sugemalimab plus chemotherapy and serplulimab plus chemotherapy exceeded the threshold. Probabilistic sensitivity analysis indicated that camrelizumab plus chemotherapy had the highest probability of being cost-effective (95%) at $27,906/QALY. In the full incremental analysis, the efficiency frontier comprised chemotherapy alone, penpulimab plus chemotherapy, camrelizumab plus chemotherapy, and pembrolizumab plus chemotherapy; camrelizumab plus chemotherapy was the preferred strategy after dominated options were excluded.

**Conclusion:**

At the selected WTP threshold, camrelizumab plus chemotherapy may be the preferred first-line strategy after strictly and extendedly dominated alternatives were excluded. This finding depends on the indirect comparisons, chemotherapy baseline, drug prices, survival extrapolation, and post-progression assumptions.

## 1 Introduction

Lung cancer (LC) remains a leading driver of the global cancer burden. In 2022, it accounted for approximately 2.5 million new cases worldwide and was responsible for an estimated 1.8 million deaths, making LC both the most commonly diagnosed cancer and the leading cause of cancer death globally (Bray et al., 2024). Beyond these, the burden is reflected in sustained loss of health, with analyses of Global Burden of Disease (GBD) 2021 indicating that global incidence, deaths, and disability-adjusted life-years (DALYs) attributable to tracheal, bronchus, and lung cancers increased between 1990 and 2021 despite declining age-standardized mortality and DALY rates (Kuang et al., 2024). In China, LC has long represented a dominant oncologic challenge, and late presentation is common. In a multicentre prospective cohort of unresectable stage IIIB/IV non-small-cell lung cancer (NSCLC) treated in China, only about 30% of newly diagnosed NSCLC cases were reported to be detected at an early stage, whereas the remaining majority were diagnosed with advanced disease (Shi et al., 2022).

NSCLC comprises approximately 80%–85% of all LCs (Chen P. et al., 2022), and lung squamous cell carcinoma (hereafter referred to as squamous non-small-cell lung cancer [sq-NSCLC]) accounts for approximately 25%–30% of NSCLC (Chen P. et al., 2022; Zhang et al., 2024). Despite advances in molecular oncology, therapeutic progress in sq-NSCLC has lagged behind that in non-squamous disease because actionable driver alterations are less common and targeted treatment opportunities are correspondingly limited (Hendriks et al., 2023). Historically, platinum-doublet chemotherapy delivered limited survival for metastatic sq-NSCLC. In KEYNOTE-407, the placebo-plus-chemotherapy control arm achieved a median overall survival (OS) of 11.3 months, highlighting the need for more effective first-line strategies (Paz-Ares et al., 2018).

Over the past decade, immune checkpoint inhibitors (ICIs) targeting programmed cell death protein 1 (PD-1) or programmed cell death ligand 1 (PD-L1) have reshaped first-line treatment paradigms for advanced NSCLC. Current international guidelines now consistently endorse immune-based combination therapy as a standard first-line option for advanced sq-NSCLC. Recommendations from the National Comprehensive Cancer Network (NCCN), the European Society for Medical Oncology (ESMO), and the Chinese Society of Clinical Oncology (CSCO) all support platinum-based chemotherapy combined with PD-1/PD-L1 blockade in patients without targetable genomic alterations (Riely et al., 2025; Wang J. et al., 2025; Hendriks et al., 2025). Notably, the 2025 CSCO guideline reflects the rapid expansion of immunotherapy options in China and recommends six ICI-based first-line regimens for advanced or metastatic driver-negative sq-NSCLC: sugemalimab, tislelizumab, penpulimab, serplulimab, camrelizumab, and pembrolizumab, each in combination with chemotherapy. The clinical efficacy and safety of these six ICI-based regimens have been evaluated in six pivotal phase three randomized controlled trials: GEMSTONE-302 (Zhou et al., 2025; Zhou C. et al., 2022), RATIONALE-307 (Wang et al., 2024), AK105-302 (Zhong et al., 2024), ASTRUM-004 (Zhou et al., 2024), CameL-Sq (Ren et al., 2022), and KEYNOTE-407 (Cheng et al., 2021; Novello et al., 2023). Collectively, these trials showed that the addition of PD-1 or PD-L1 blockade to chemotherapy improved progression-free survival (PFS) and OS compared with chemotherapy alone. The principal study characteristics, efficacy outcomes, and safety findings are summarized systematically in Supplementary Tables S1 and S2.

However, the rapid increase in available regimens has introduced a new challenge: how to identify the strategy that delivers the greatest health benefit for the resources consumed. Although these agents have improved survival, they differ in acquisition cost, administration pattern, toxicity burden and subsequent resource use. This issue is especially relevant in China, where LC accounts for substantial medical expenditure and where the affordability of innovative anticancer therapies remains central to reimbursement and access decisions. Cost-effectiveness analysis (CEA), typically expressed as an incremental cost-effectiveness ratio (ICER) per quality-adjusted life-year (QALY) gained, provides a structured framework to evaluate whether incremental benefits justify incremental costs under a specified willingness-to-pay (WTP) threshold. Evidence from earlier CEA conducted in China is valuable yet incomplete. Most published studies in advanced sq-NSCLC have evaluated only individual ICI regimens against chemotherapy alone, such as analyses focused separately on pembrolizumab, camrelizumab, penpulimab or other single strategies (Zhang et al., 2023; Liang et al., 2023; Wang L. et al., 2025; Xiang et al., 2024). Other comparative studies were conducted earlier and therefore did not incorporate newer regimens, updated survival data, or the current treatment landscape (Zhao et al., 2023; Lang et al., 2025). This gap has become more important for several reasons. First, new regimens, particularly penpulimab and serplulimab, have entered routine clinical consideration following positive phase III results (Zhong et al., 2024; Zhou et al., 2024). Second, the prices of ICIs in China have changed over time owing to reimbursement negotiation, market competition and broader policy adjustments, potentially altering earlier conclusions regarding economic value. Third, the methodological framework for pharmacoeconomic evaluation in China has also evolved. The China Guidelines for Pharmacoeconomic Evaluations (2025) updated the WTP benchmark and now recommend a threshold of up to two times gross domestic product (GDP) *per capita*, replacing the more permissive threshold range commonly used in earlier analyses (Jing and Liu, 2025). These developments together indicate that prior evaluations may no longer reflect current clinical practice, market conditions or decision thresholds.

In this context, the present study aims to conduct an updated economic evaluation of six ICI-based first-line therapies for advanced sq-NSCLC in China, integrating network meta-analysis (NMA) derived comparative effectiveness with current cost inputs and applying the updated WTP threshold recommendations from China Guidelines for Pharmacoeconomic Evaluations (2025). This analysis is intended to inform clinical decision-making and support more efficient allocation of LC treatment resources in China.

## 2 Materials and methods

### 2.1 Study design and overview

This study conducted a comprehensive CEA of six ICI-based first-line treatment strategies for patients with advanced sq-NSCLC: sugemalimab, tislelizumab, penpulimab, serplulimab, camrelizumab, and pembrolizumab, each combined with chemotherapy. The chemotherapy backbone for each treatment strategy was modelled according to the corresponding pivotal trial. Serplulimab was combined with nab-paclitaxel and carboplatin, whereas sugemalimab, tislelizumab, penpulimab, camrelizumab, and pembrolizumab were combined with paclitaxel and carboplatin. Trial-specific dosing schedules, treatment durations, and drug costs were incorporated into the model. The overall analytical framework is presented in Figure 1. This study was designed and reported in accordance with the Consolidated Health Economic Evaluation Reporting Standards (CHEERS 2022) (Husereau et al., 2022), with detailed compliance provided in Supplementary Table S3.

**FIGURE 1:**
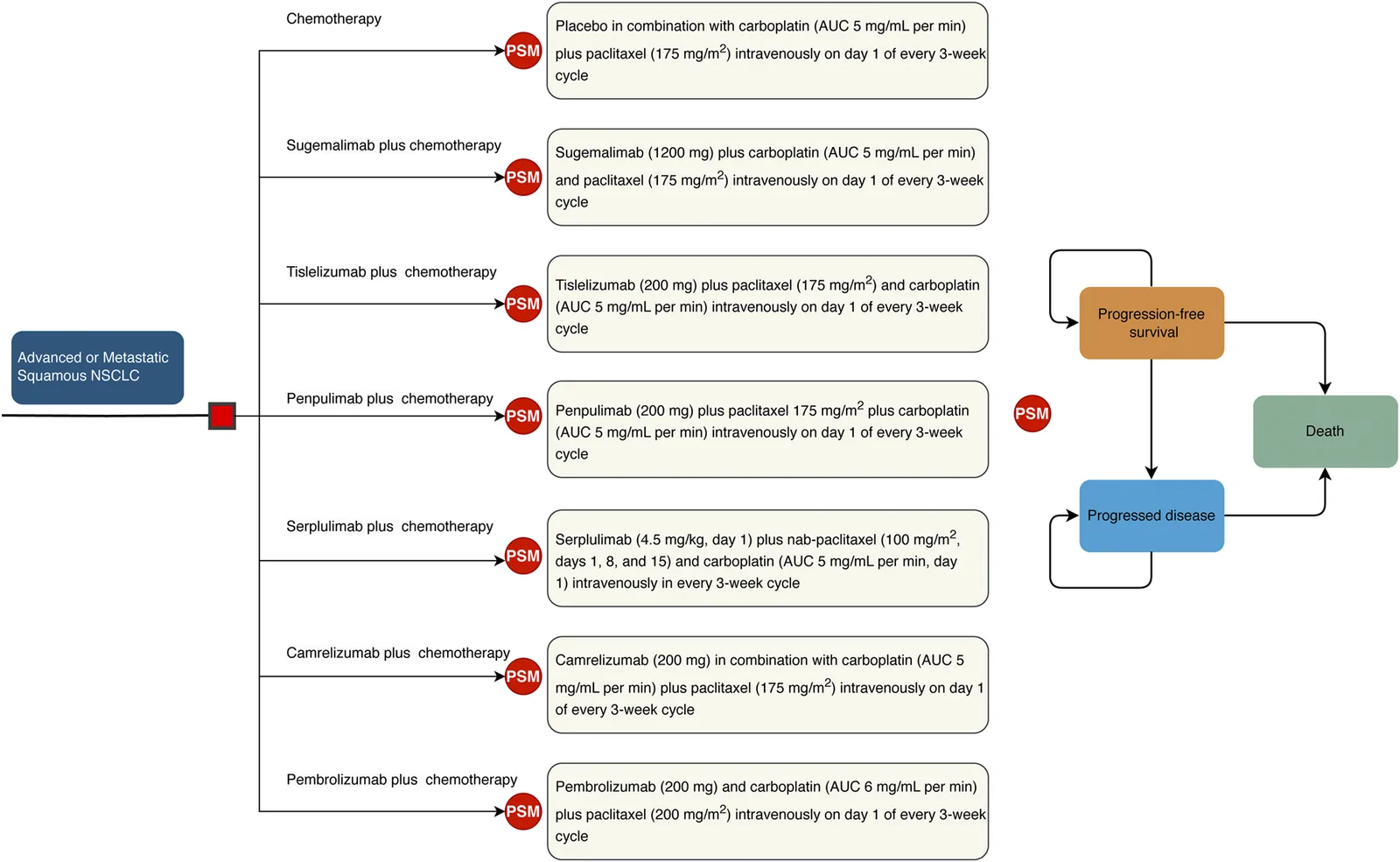
Partitioned survival model structure. Abbreviation: PSM = partitioned survival model. AUC = area under the curve.

### 2.2 Cost-effectiveness analysis framework

A model-based economic evaluation was performed from the perspective of the Chinese healthcare system. The primary outcome measures included total costs, life-years (LYs), QALYs, ICERs, and incremental net monetary benefits (INMBs). A full incremental CEA was also conducted by ordering all mutually exclusive strategies according to total cost, excluding strictly and extendedly dominated options, and calculating sequential ICERs for strategies remaining on the cost-effectiveness frontier.

#### 2.2.1 Model construction

A partitioned survival model (PSM) was developed to simulate disease progression and estimate long-term clinical and economic outcomes. The model consisted of three mutually exclusive health states: PFS, progressed disease (PD), and death. All patients entered the model in the PFS state and transitioned across health states over time. The proportions of patients in each health state at any given time point were derived directly from OS and PFS curves, consistent with standard PSM methodology. The PD state was calculated as the difference between OS and PFS, while death was defined as an absorbing state. A lifetime time horizon was adopted to fully capture all relevant costs and health outcomes, assuming that more than 99% of the cohort would eventually transition to death. The model cycle length was set at 21 days to align with the clinical treatment cycle of ICIs combined with chemotherapy. In accordance with the China Guidelines for Pharmacoeconomic Evaluations (2025), both costs and health outcomes were discounted at an annual rate of 4.5% (Jing and Liu, 2025).

#### 2.2.2 Clinical data and survival modelling

Clinical efficacy data, including PFS and OS, were obtained from six published randomized phase III trials (Zhou et al., 2025; Zhou C. et al., 2022; Wang et al., 2024; Zhong et al., 2024; Zhou et al., 2024; Ren et al., 2022; Cheng et al., 2021). The Kaplan–Meier (KM) curves for the chemotherapy reference were derived from the sq-NSCLC subgroup of GEMSTONE-302 (Zhou et al., 2025; Zhou C. et al., 2022). This subgroup was selected because it enrolled Chinese patients with treatment-naive advanced sq-NSCLC, used paclitaxel plus carboplatin as the chemotherapy backbone, and provided relatively mature long-term follow-up compared with the other available trials. These features improved the clinical relevance of the baseline survival curves to the target population and the Chinese healthcare setting. The reconstructed chemotherapy-arm OS and PFS curves from this subgroup were therefore used as the common baseline for the six modelled ICI-based regimens (Supplementary Figure S1). Because individual patient data (IPD) were not directly available, KM curves were digitized using WebPlotDigitizer (https://apps.automeris.io/wpd4/index.zh_CN.html). Subsequently, pseudo-IPD were reconstructed using established statistical algorithms implemented in R software, following the method described by Guyot et al. (Guyot et al., 2012) Parametric survival models, including exponential, Weibull, gamma, Gompertz, log-normal, log-logistic, and generalized gamma distributions, were fitted separately to the reconstructed OS and PFS data from the chemotherapy arm of the GEMSTONE-302 sq-NSCLC subgroup. Model selection was based on the Akaike Information Criterion (AIC) and Bayesian Information Criterion (BIC), with lower values indicating better statistical fit. Visual agreement between the fitted curves and reconstructed KM data was also examined. The log-logistic distribution yielded the lowest AIC and BIC values for both OS and PFS and adequately reproduced the observed survival patterns; it was therefore selected for the base-case analysis. The fitted curves are presented in Supplementary Figure S2, the model-selection statistics are reported in Supplementary Table S4, and the corresponding log-logistic scale and shape parameters are provided in Table 1.

**TABLE 1:** Key model inputs, uncertainty ranges, distributions, and data sources.

Parameters	Baseline	Range		Distribution	Source
		Minimum	Maximum		
Survival model for chemotherapy					
Log-logistic OS survival model with chemotherapy	shape = 1.50732; scale = 13.21761			Reconstructed GEMSTONE-302 sq-NSCLC subgroup	
Log-logistic PFS survival model with chemotherapy	shape = 2.61844; scale = 4.34907			Reconstructed GEMSTONE-302 sq-NSCLC subgroup	
Drug acquisition costs ($)					
Sugemalimab per 600 mg	1,732.05	1,385.64	2,078.46	Gamma	(The big data service platform for China’s health industry, 2026)
Tislelizumab per 100 mg	175.45	140.36	210.54	Gamma	(The big data service platform for China’s health industry, 2026)
Penpulimab per 100 mg	126.11	100.89	151.33	Gamma	(The big data service platform for China’s health industry, 2026)
Serplulimab per 100 mg	782.12	625.69	938.54	Gamma	(The big data service platform for China’s health industry, 2026)
Camrelizumab per 200 mg	360.64	288.51	432.76	Gamma	(The big data service platform for China’s health industry, 2026)
Pembrolizumab per 100 mg	2,507.87	2,006.29	3,009.44	Gamma	(The big data service platform for China’s health industry, 2026)
Carboplatin per 50 mg	7.00	5.60	8.40	Gamma	(The big data service platform for China’s health industry, 2026)
Paclitaxel per 30 mg	5.32	4.25	6.38	Gamma	(The big data service platform for China’s health industry, 2026)
Nab-paclitaxel per 100 mg	48.20	38.56	57.84	Gamma	(The big data service platform for China’s health industry, 2026)
Other medical costs ($)					
Cost of intravenous injection	1.78	1.60	2.21	Gamma	(Shao et al., 2022; You et al., 2025)
Cost of Care	3.89	3.07	4.61	Gamma	(Shao et al., 2022; You et al., 2025)
Cost of Bed	6.81	5.38	8.08	Gamma	(Shao et al., 2022; You et al., 2025)
Cost of CT	59.99	47.43	71.14	Gamma	(Shao et al., 2022; You et al., 2025)
Cost of Blood routine test	3.24	2.57	3.85	Gamma	(Shao et al., 2022; You et al., 2025)
Cost of Biochemical test	48.52	38.36	57.55	Gamma	(Shao et al., 2022; You et al., 2025)
Cost of Routine urine test	0.65	0.52	0.77	Gamma	(Shao et al., 2022; You et al., 2025)
Cost of Best supportive care per cycle	352.27	281.81	422.72	Gamma	(Wang et al., 2025b)
Cost of End-of-life per patient	2,398.56	1,918.85	2,878.27	Gamma	(Shao et al., 2022; You et al., 2025)
AE management costs ($)					
Cost of Leukopenia	119.17	52.34	366.42	Gamma	(Shao et al., 2022)
Cost of Neutropenia	119.17	52.34	366.42	Gamma	(Shao et al., 2022)
Cost of Pneumonitis	1,679.52	1,343.62	2,015.43	Gamma	(Zhao et al., 2023)
Cost of Thrombocytopenia	1,533.96	1,227.17	1,840.72	Gamma	(Wang et al., 2025b)
Cost of Anemia	143.78	109.30	163.96	Gamma	(Shao et al., 2022)
Time duration of grade 3–5 AEs/days					
Time duration of Anemia	51.20	40.96	61.44	Normal	(Zhao et al., 2023; Rui et al., 2022)
Time duration of Leukopenia	4.50	3.60	5.40	Normal	(Zhao et al., 2023; Rui et al., 2022)
Time duration of Neutropenia	6.40	5.12	7.68	Normal	(Zhao et al., 2023; Rui et al., 2022)
Time duration of Thrombocytopenia	47.29	37.83	56.75	Normal	(Shao et al., 2022)
Time duration of Pneumonitis	21.00	16.80	25.20	Normal	(Shao et al., 2022)
Health-state utilities					
Utility of PFS	0.80	0.64	0.97	Beta	(Liang et al., 2023; Wang et al., 2025b; Nafees et al., 2017; Zheng et al., 2024)
Utility of PD	0.32	0.26	0.39	Beta	(Liang et al., 2023; Wang et al., 2025b; Nafees et al., 2017; Zheng et al., 2024)
AE disutilities					
Disutility of Pneumonitis	0.05	0.04	0.06	Beta	(Zhao et al., 2023; Chen et al., 2022b)
Disutility of Leukopenia	0.20	0.16	0.24	Beta	(Nafees et al., 2017; Cai et al., 2025; Liu et al., 2025)
Disutility of Neutropenia	0.20	0.16	0.24	Beta	(Nafees et al., 2017; Cai et al., 2025; Liu et al., 2025)
Disutility of Thrombocytopenia	0.11	0.09	0.13	Beta	(Wang et al., 2025b; Liu et al., 2025)
Disutility of Anemia	0.07	0.06	0.09	Beta	(Wang et al., 2025b)
Other model parameters					
Body area surface, m^2^	1.72	1.38	2.06	Normal	(Liu et al., 2025)
Weight/kg	65.00	32.50	97.50	Normal	(Xiang et al., 2024; Zhou et al., 2022b)
Creatinine clearance/mL min^−1^	70.00	35.00	105.00	Normal	(You et al., 2025; Zhang et al., 2025)
Discount rate (%)	4.5%	3.0%	5.0%	Beta	(Jing and Liu, 2025)

Treatment‐specific incidences of grade ≥3 AEs are reported in Supplementary Table S6. Abbreviation: AE, adverse event; PFS, progression-free survival; PD, progressed disease; CT, computed tomography.

#### 2.2.3 Network meta-analysis

A NMA was conducted to estimate the comparative effects of the six ICI-based regimens on OS and PFS in the absence of direct head-to-head trials. All eligible comparisons evaluated an ICI plus platinum-based chemotherapy against a chemotherapy-containing control, thereby forming a star-shaped network connected through the chemotherapy node. The structure of the evidence network is presented in Supplementary Figure S3.

Published HRs and corresponding 95% confidence intervals (CIs) for OS and PFS were extracted. For GEMSTONE-302, only data from the sq-NSCLC subgroup were used in the present analysis, rather than results from the overall trial population that included both squamous and non-squamous histology subgroups. The treatment-effect estimates for sugemalimab were also obtained from the squamous subgroup. This selection improved consistency between the GEMSTONE-302 population and the exclusively sq-NSCLC population evaluated in the economic model. Detailed trial populations, data cutoffs, chemotherapy backbones, and efficacy estimates used in the analysis are reported in Supplementary Tables S1 and S2.

HRs were analyzed on the logarithmic scale, and their standard errors were derived from the reported 95% CIs (Supplementary Table S5). Chemotherapy was used as the reference treatment. Relative effects between active ICI-based regimens were estimated indirectly through their respective comparisons with chemotherapy. Because each active regimen was primarily informed by a single pivotal trial and the evidence network contained no closed loops, within-comparison between-study heterogeneity and statistical inconsistency between direct and indirect evidence could not be formally estimated. No individual patient data-based population adjustment, matching-adjusted indirect comparison, meta-regression, or subgroup-adjusted NMA was undertaken. The NMA therefore did not formally adjust for cross-trial differences in geographic setting, disease stage, ECOG performance status, PD-L1 distribution, chemotherapy backbone, follow-up duration, treatment crossover, or subsequent anticancer therapy. These characteristics and potential sources of clinical heterogeneity are descriptively summarized in Supplementary Table S2 to support interpretation of the indirect comparative estimates.

The NMA-derived HRs were subsequently applied to the parametric chemotherapy survival curves fitted to the GEMSTONE-302 sq-NSCLC subgroup under the proportional hazards assumption (Supplementary Figure S4). These curves determined the proportions of patients in the progression-free, progressed-disease, and death states over time.

#### 2.2.4 Costs and utilities

Direct medical costs included drug acquisition, treatment administration, management of adverse events (AEs), best supportive care after disease progression, routine medical examinations, and end-of-life care. After disease progression, patients were assumed to receive best supportive care. The corresponding cost was applied during each cycle spent in the PD state. Specific subsequent anticancer therapies and treatment switching were not modelled because comparable regimen-specific data were not consistently available across the included trials. The cost of best supportive care was varied within its prespecified range in the deterministic and probabilistic sensitivity analyses. To ensure temporal comparability, all cost inputs were adjusted to 2025 price levels using the Chinese Consumer Price Index (CPI). After inflation adjustment, costs were converted into United States dollars (USD) using an exchange rate of 1 USD = 7.14 CNY in 2025. Drug doses, administration schedules, chemotherapy backbones, and treatment durations were based on the corresponding pivotal clinical trials. Drug acquisition costs were obtained from the drug winning-bid price database of Yaozh (https://www.yaozh.com/) (The big data service platform for China's health industry, 2026). When multiple eligible winning-bid prices were available, the median value was used as the base-case unit price to reduce the influence of extreme regional observations. All other cost inputs, including treatment administration, medical examinations, management of AEs, best supportive care, and end-of-life care, were obtained from published literature. Health-state utility values of 0.80 for PFS and 0.32 for PD were obtained from previously published economic evaluations of advanced sq-NSCLC (Liang et al., 2023; Wang L. et al., 2025; Nafees et al., 2017; Zheng et al., 2024). These values have been commonly used in cost-effectiveness analyses of immunotherapy-based treatments and were adopted because comparable trial-based utility data were not consistently reported across all included regimens. A utility value of 0 was assigned to death. Disutility associated with AEs was incorporated. Only grade 3 or higher AEs with an incidence exceeding 5% were included in the model. This approach is consistent with previous oncology CEAs that have focused on frequent severe toxicities because these events are expected to account for the majority of AE-related costs and utility losses (Yoo et al., 2023; Su et al., 2021; Kuznik et al., 2022; Konidaris et al., 2021). In the base-case analysis, the costs associated with AEs were applied during the first model cycle. The incidence, duration, management cost, and utility decrement of each included AE were obtained from the pivotal trials and published literature (Table 1; Supplementary Table S6). Uncertainty in AE-related parameters was assessed in the sensitivity analyses.

#### 2.2.5 Willingness-to-pay threshold

The WTP threshold was set at $27,906 per QALY gained, corresponding to two times the GDP *per capita* in China, in accordance with the China Guidelines for Pharmacoeconomic Evaluations (2025) (Jing and Liu, 2025). Interventions with ICERs below this threshold were considered cost-effective.

### 2.3 Sensitivity analyses

To evaluate the impact of parameter uncertainty on model outcomes, one-way sensitivity analyses were conducted using the reported 95% CIs where available. When 95% CIs were unavailable, parameters were varied from 80% to 120% of their respective base-case values. The results were presented as tornado diagrams to identify the parameters with the greatest influence on the model outcomes. A probabilistic sensitivity analysis (PSA) was performed to simultaneously assess the uncertainty of all model parameters. Appropriate probability distributions were assigned to each parameter, including gamma distributions for costs and beta distributions for utility values and probabilities. A total of 1,000 Monte Carlo simulations were conducted to generate distributions of incremental costs and QALYs. The results were visualized using scatterplots on the cost-effectiveness plane and cost-effectiveness acceptability curve (CEAC), illustrating the probability of each treatment being cost-effective across a range of WTP thresholds.

### 2.4 Scenario analyses

To further evaluate the robustness of the model under alternative assumptions, several scenario analyses were conducted. First, to reflect potential impacts of national pharmaceutical pricing policies, ICI acquisition prices were set at 90%, 75%, 50%, and 25% of their respective base-case values. Second, alternative time horizons were explored by limiting survival to 10 years and 8 years, respectively, to assess the influence of long-term extrapolation on cost-effectiveness outcomes. Fourth, the chemotherapy-arm OS and PFS curves from the CameL-Sq trial were used as an alternative common baseline survival reference. CameL-Sq was selected because it enrolled Chinese patients with advanced sq-NSCLC and used paclitaxel plus carboplatin, consistent with the chemotherapy backbone of the GEMSTONE-302 sq-NSCLC subgroup. The published KM curves were digitized, pseudo-individual patient data were reconstructed, and parametric survival distributions were fitted using the same procedures and model-selection criteria as in the base-case analysis. The NMA-derived treatment-specific HRs were then applied to the alternative chemotherapy survival curves, and costs, LYs, QALYs, and ICERs were recalculated. Fifth, uncertainty related to the selection of the parametric survival distributions was evaluated using the second-best-fitting models. In Scenario 5, the log-normal distribution was used for OS and the gamma distribution was used for PFS. These scenario analyses were designed to test structural assumptions and enhance the generalizability of the findings.

All data processing, statistical analyses, and graphical visualizations were performed using R software (version 4.4.1; R Foundation for Statistical Computing, Vienna, Austria) and Microsoft Excel for Mac (version 16.101.3 [25100321]; Microsoft Corporation, Redmond, WA, United States). The packages used for this study included ‘ggplot2’ (version 3.5.1), ‘tidyverse’ (version 2.0.0), ‘flexsurv’ (version 2.3.2) and ‘survHE’ (version 2.0.5) (Baio, 2020; Jackson, 2016).

## 3 Results

### 3.1 Base-case analysis

The results of the base-case analysis are presented in Table 2, with a detailed breakdown of costs and health outcomes for each treatment strategy in Supplementary Table S7. Chemotherapy was associated with the lowest total cost ($12,227.47), with the lowest health benefit (1.58 LYs, 0.71 QALYs) (Figure 2). All six ICI-based first-line regimens increased both LYs and QALYs relative to chemotherapy, although their economic outcomes differed substantially. Pembrolizumab plus chemotherapy yielded the highest QALYs (2.07) and also incurred the highest total cost ($108,217.87), resulting in an ICER of $70,913.04/QALY versus chemotherapy (Figure 2). Camrelizumab plus chemotherapy produced 1.80 QALYs at a total cost of $26,750.87, with an ICER of $13,361.50/QALY, whereas penpulimab plus chemotherapy generated 1.65 QALYs at $24,460.85 (ICER: $13,064.23/QALY) (Figure 2). Tislelizumab plus chemotherapy also demonstrated relatively favorable economic outcomes, with QALY of 1.39 and an ICER of $14,022.95/QALY. By contrast, sugemalimab plus chemotherapy and serplulimab plus chemotherapy were associated with substantially higher costs ($76,492.08 and $46,852.58, respectively) and markedly higher ICERs ($61,853.91/QALY and $66,362.71/QALY), despite improved QALYs.

**TABLE 2:** Summary of cost and outcome results in the base-case analysis and the scenario analysis.

Treatment	Costs	LYs	QALYs	ICER vs. chemotherapy ($/LY)	ICER vs. chemotherapy ($/QALY)	INMB vs. chemotherapy ($)
Base-case analysis results						
Chemotherapy alone	12,227.47	1.58	0.71	NA	NA	NA
Sugemalimab plus chemotherapy	76,492.08	3.09	1.75	42,700.94	61,853.91	−35,271.00
Tislelizumab plus chemotherapy	21,661.26	2.71	1.39	8,339.44	14,022.95	9,339.68
Penpulimab plus chemotherapy	24,460.85	3.42	1.65	6,667.84	13,064.23	13,897.87
Serplulimab plus chemotherapy	46,852.58	2.55	1.24	35,746.03	66,362.71	−20,065.00
Camrelizumab plus chemotherapy	26,750.87	3.46	1.80	7,743.34	13,361.50	15,809.27
Pembrolizumab plus chemotherapy	108,217.87	4.16	2.07	37,250.26	70,913.04	−58,215.85
Scenario 1: ICI acquisition prices set at 90%, 75%, 50%, and 25% of the base-case values						
Sugemalimab plus chemotherapy						
90% Sugemalimab	70,656.22	3.09	1.75	38,823.27	56,236.97	−29,435.14
75% Sugemalimab	62,577.10	3.09	1.75	33,455.06	48,460.91	−21,356.02
50% Sugemalimab	47,312.77	3.09	1.75	23,312.60	33,769.18	−6,091.69
25% Sugemalimab	32,723.12	3.09	1.75	13,618.44	19,726.82	8,497.96
Tislelizumab plus chemotherapy						
90% Tislelizumab	21,165.86	2.71	1.39	7,901.51	13,286.56	9,835.07
75% Tislelizumab	20,422.77	2.71	1.39	7,244.61	12,181.98	10,578.17
50% Tislelizumab	19,184.28	2.71	1.39	6,149.79	10,341.01	11,816.66
25% Tislelizumab	17,945.78	2.71	1.39	5,054.97	8,500.04	13,055.16
Penpulimab plus chemotherapy						
90% Penpulimab	24,093.80	3.42	1.65	6,467.78	12,672.25	14,264.92
75% Penpulimab	23,543.23	3.42	1.65	6,167.69	12,130.01	14,717.00
50% Penpulimab	22,625.61	3.42	1.65	5,667.54	11,104.34	15,733.11
25% Penpulimab	21,707.98	3.42	1.65	5,167.38	10,124.40	16,650.73
Serplulimab plus chemotherapy						
90% Serplulimab	43,896.54	2.55	1.24	32,694.30	60,697.16	−17,108.96
75% Serplulimab	39,462.49	2.55	1.24	28,116.70	52,466.36	−12,749.16
50% Serplulimab	32,072.40	2.55	1.24	20,487.37	38,034.93	−5,284.82
25% Serplulimab	24,682.31	2.55	1.24	12,858.04	23,871.04	2,105.26
Camrelizumab plus chemotherapy						
90% Camrelizumab	26,123.49	3.46	1.80	7,408.85	12,784.32	16,436.65
75% Camrelizumab	25,257.13	3.46	1.80	6,946.94	12,036.15	17,179.81
50% Camrelizumab	23,813.19	3.46	1.80	6,177.08	10,658.84	18,746.95
25% Camrelizumab	22,369.25	3.46	1.80	5,407.23	9,330.42	20,190.89
Pembrolizumab plus chemotherapy						
90% Pembrolizumab	99,911.04	4.16	2.07	34,026.70	64,776.35	−49,909.02
75% Pembrolizumab	87,450.80	4.16	2.07	29,191.34	55,571.33	−37,448.77
50% Pembrolizumab	66,683.72	4.16	2.07	21,132.42	40,229.63	−16,681.70
25% Pembrolizumab	45,916.65	4.16	2.07	13,073.50	25,027.14	3,875.25
Scenario 2: 10 years						
Chemotherapy	12,225.68	1.58	0.71	NA	NA	NA
Sugemalimab	75,063.88	2.89	1.64	47,972.06	67,549.93	−36,878.69
Tislelizumab	20,646.71	2.59	1.33	8,343.44	13,650.20	8,794.64
Penpulimab	22,572.10	3.15	1.54	6,587.14	12,443.36	12,856.89
Serplulimab	46,128.87	2.46	1.20	38,683.73	70,029.78	−20,393.19
Camrelizumab	24,535.59	3.15	1.66	7,837.08	13,067.83	13,977.57
Pembrolizumab	105,221.98	3.73	1.89	43,312.02	79,239.12	−60,245.37
Scenario 3: 8 years						
Chemotherapy	12,223.44	1.58	0.71	NA	NA	NA
Sugemalimab	73,709.46	2.70	1.55	55,123.18	73,860.68	−38,255.41
Tislelizumab	19,595.58	2.44	1.27	8,574.20	13,280.48	8,118.78
Penpulimab	20,935.34	2.92	1.45	6,529.10	11,786.90	11,913.90
Serplulimab	45,204.70	2.33	1.15	44,222.30	75,787.72	−20,837.14
Camrelizumab	22,898.82	2.92	1.55	8,000.46	12,728.61	12,729.15
Pembrolizumab	102,908.00	3.39	1.75	50,102.17	87,795.94	−61,860.41
Scenario 4: the chemotherapy-arm survival curves from CameL-Sq						
Chemotherapy	11,766.13	1.52	0.72	NA	NA	NA
Sugemalimab	76,604.94	2.56	1.63	61,913.38	70,870.40	−39,307.82
Tislelizumab	18,718.24	2.26	1.28	9,396.44	12,308.39	8,809.95
Penpulimab	19,827.74	2.71	1.45	6,781.81	10,966.61	12,452.24
Serplulimab	46,557.08	2.14	1.15	55,458.72	81,351.67	−22,856.64
Camrelizumab	25,758.31	3.33	1.81	7,733.36	13,810.79	14,280.34
Pembrolizumab	106,575.02	3.35	1.83	51,827.22	81,771.86	−62,453.79
Scenario 5: the second-best-fitting models						
Chemotherapy	12,644.81	1.64	0.73	NA	NA	NA
Sugemalimab	78,519.56	3.17	1.64	43,172.56	72,428.17	−40,493.73
Tislelizumab	22,162.52	2.79	1.34	8,326.49	15,461.18	7,660.88
Penpulimab	25,178.46	3.52	1.61	6,681.60	14,269.00	11,978.53
Serplulimab	48,371.05	2.62	1.22	36,397.39	72,072.92	−21,893.36
Camrelizumab	27,236.46	3.52	1.73	7,778.56	14,560.77	13,373.54
Pembrolizumab	111,895.73	4.36	2.00	36,539.53	77,942.75	−63,715.92

Abbreviation: LY, life-year; QALY, quality-adjusted life-year; ICER, incremental cost-effectiveness ratio; INMB, incremental net monetary benefits.

**FIGURE 2:**
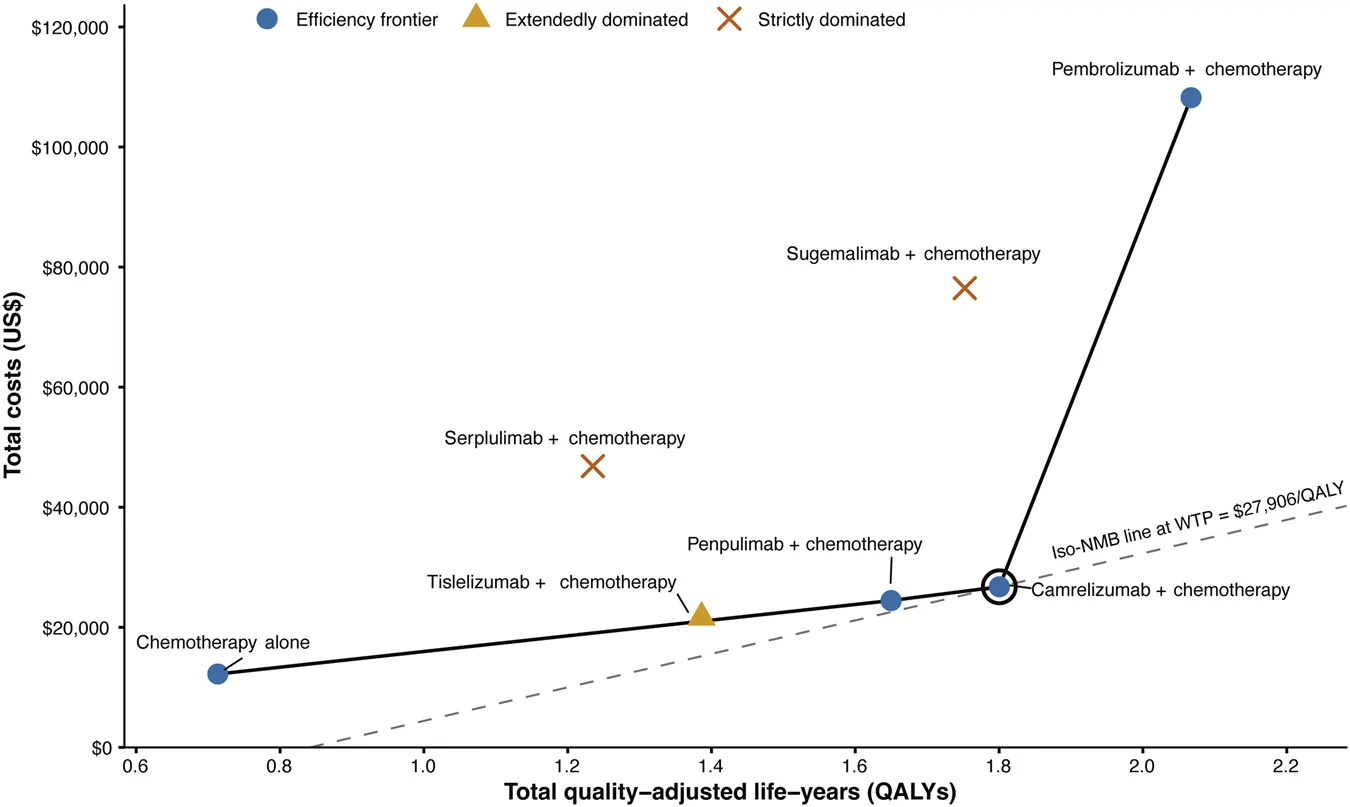
Cost-effectiveness analysis for chemotherapy alone and six immune checkpoint inhibitor-based first-line regimens. Each point represents the total cost and total QALYs associated with a treatment strategy. The solid line connects the strategies remaining on the cost-effectiveness efficiency frontier. The dashed line represents an iso-NMB line with a slope equal to the WTP threshold of $27,906 per QALY and passing through camrelizumab plus chemotherapy, the strategy with the highest net monetary benefit at this threshold. Abbreviations: ICER = incremental cost-effectiveness ratio; QALY = quality-adjusted life-year; WTP = willingness-to-pay; iso-NMB = iso–net monetary benefit.

A full incremental cost-effectiveness analysis was conducted across all mutually exclusive strategies, and the resulting cost-effectiveness efficiency frontier is presented in Figure 2. Serplulimab plus chemotherapy and sugemalimab plus chemotherapy were strictly dominated because they were more costly and less effective than other available strategies, whereas tislelizumab plus chemotherapy was subject to extended dominance. The efficiency frontier comprised chemotherapy alone, penpulimab plus chemotherapy, camrelizumab plus chemotherapy, and pembrolizumab plus chemotherapy. Compared with chemotherapy alone, penpulimab plus chemotherapy had a sequential ICER of $13,054.86/QALY. Camrelizumab plus chemotherapy provided additional health benefit relative to penpulimab plus chemotherapy, with a sequential ICER of $15,278.56/QALY, which remained below the WTP threshold of $27,906/QALY. Pembrolizumab plus chemotherapy yielded the greatest number of QALYs but had a sequential ICER of approximately $305,489.63/QALY compared with camrelizumab plus chemotherapy. Figure 2 visually illustrates the strategies remaining on the efficiency frontier and the dominance status of the excluded alternatives. Detailed numerical results are provided in Supplementary Table S8.

### 3.2 Sensitivity analyses

In the one-way sensitivity analysis, the model outcomes were driven primarily by the utility values for PFS, the utility of PD, and the acquisition costs of ICIs, whereas other parameters had comparatively limited effects on the ICERs (Figures 3, 4). Across the high-cost regimens, including pembrolizumab plus chemotherapy, sugemalimab plus chemotherapy, and serplulimab plus chemotherapy, variations in these key parameters produced marked changes in ICERs (Figure 3). However, the resulting ICERs remained consistently above the WTP threshold of $27,906/QALY. By contrast, for camrelizumab plus chemotherapy, penpulimab plus chemotherapy, and tislelizumab plus chemotherapy, although ICERs were also most sensitive to drug prices, cost of best supportive care, and health-state utilities, all plausible parameter variations kept the ICERs below the same threshold (Figure 4). Notably, body weight had a particularly large effect on serplulimab plus chemotherapy. Overall, these findings suggest that the base-case results were structurally stable and that the economic conclusions were mainly determined by immunotherapy drug costs and utility assumptions.

**FIGURE 3:**
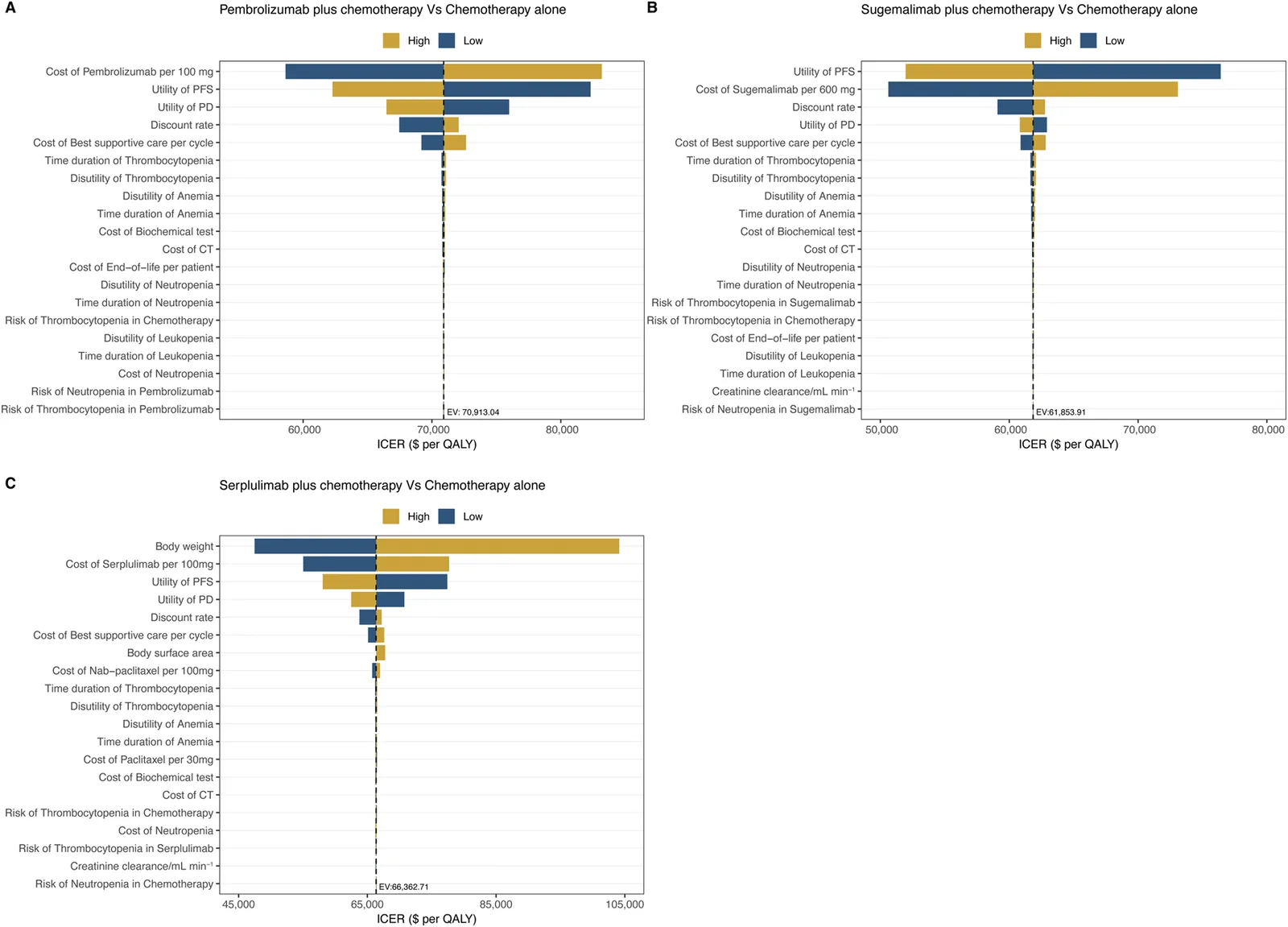
Tornado plot generated in the deterministic sensitivity analysis for pembrolizumab plus chemotherapy **(A)**, sugemalimab plus chemotherapy **(B)**, and serplulimab plus chemotherapy **(C)** Only the top 20 influential parameters are presented. Abbreviation: OS = overall survival; PFS = progression-free survival; PD = progressed disease; CT = computed tomography; QALY = quality‐adjusted life‐year; ICER = incremental cost-effectiveness ratio; EV = Expected Value.

**FIGURE 4:**
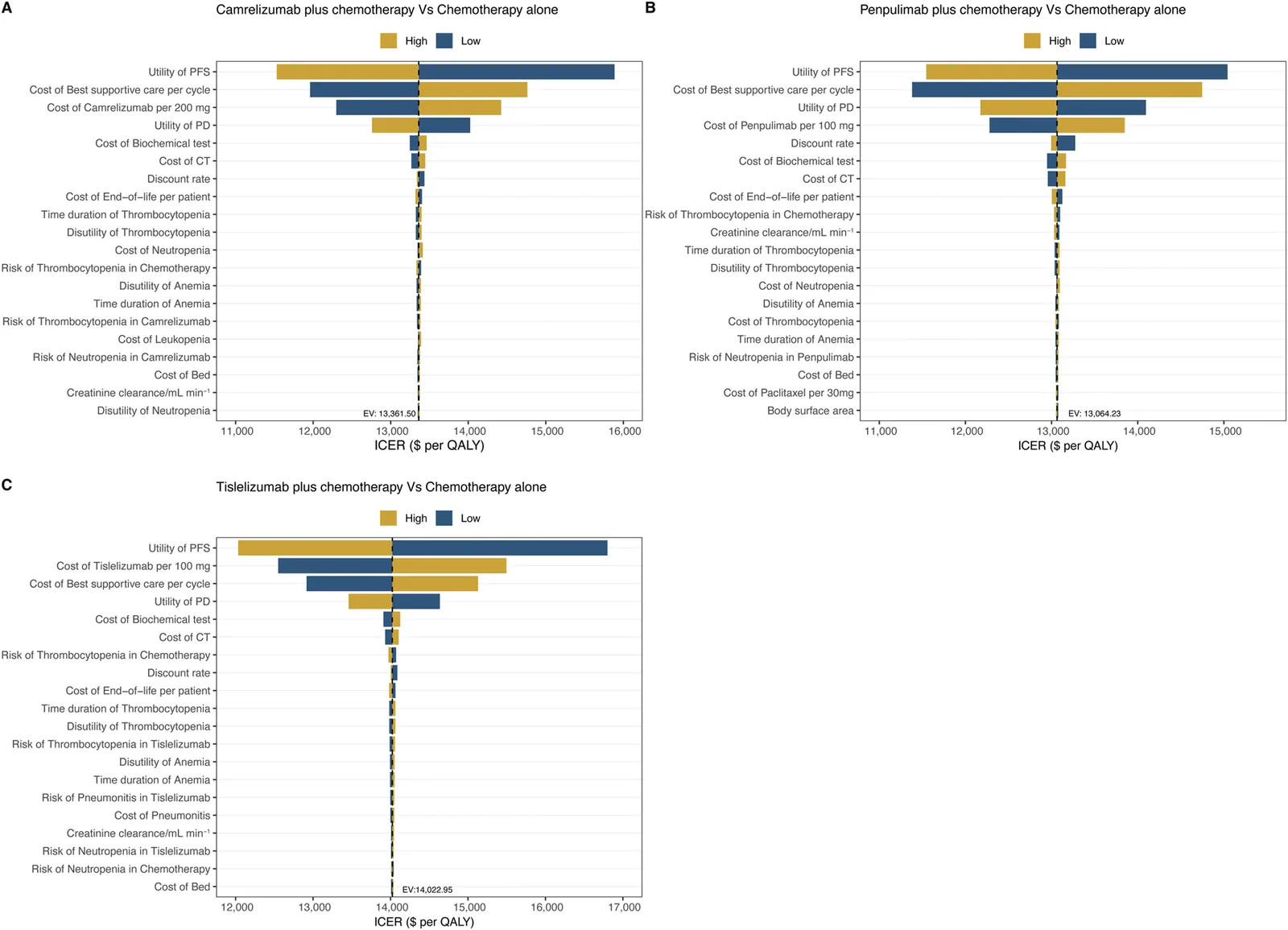
Tornado plot generated in the deterministic sensitivity analysis for camrelizumab plus chemotherapy **(A)**, penpulimab plus chemotherapy **(B)**, and tislelizumab plus chemotherapy **(C)**. Only the top 20 influential parameters are presented. Abbreviation: OS = overall survival; PFS = progression-free survival; PD = progressed disease; CT = computed tomography; QALY = quality-adjusted life-year; ICER = incremental cost-effectiveness ratio; EV = Expected Value.

In PSA, all six ICI-based regimens were associated with positive incremental health benefits versus chemotherapy alone across nearly all simulations (Figure 5A). The simulated estimates were concentrated mainly in the northeast quadrant of the cost-effectiveness scatter plot. Relative to the WTP threshold of $27,906/QALY, pembrolizumab plus chemotherapy, sugemalimab plus chemotherapy and serplulimab plus chemotherapy were distributed predominantly above the threshold line, whereas camrelizumab plus chemotherapy, penpulimab plus chemotherapy, and tislelizumab plus chemotherapy were distributed mainly below it.

**FIGURE 5:**
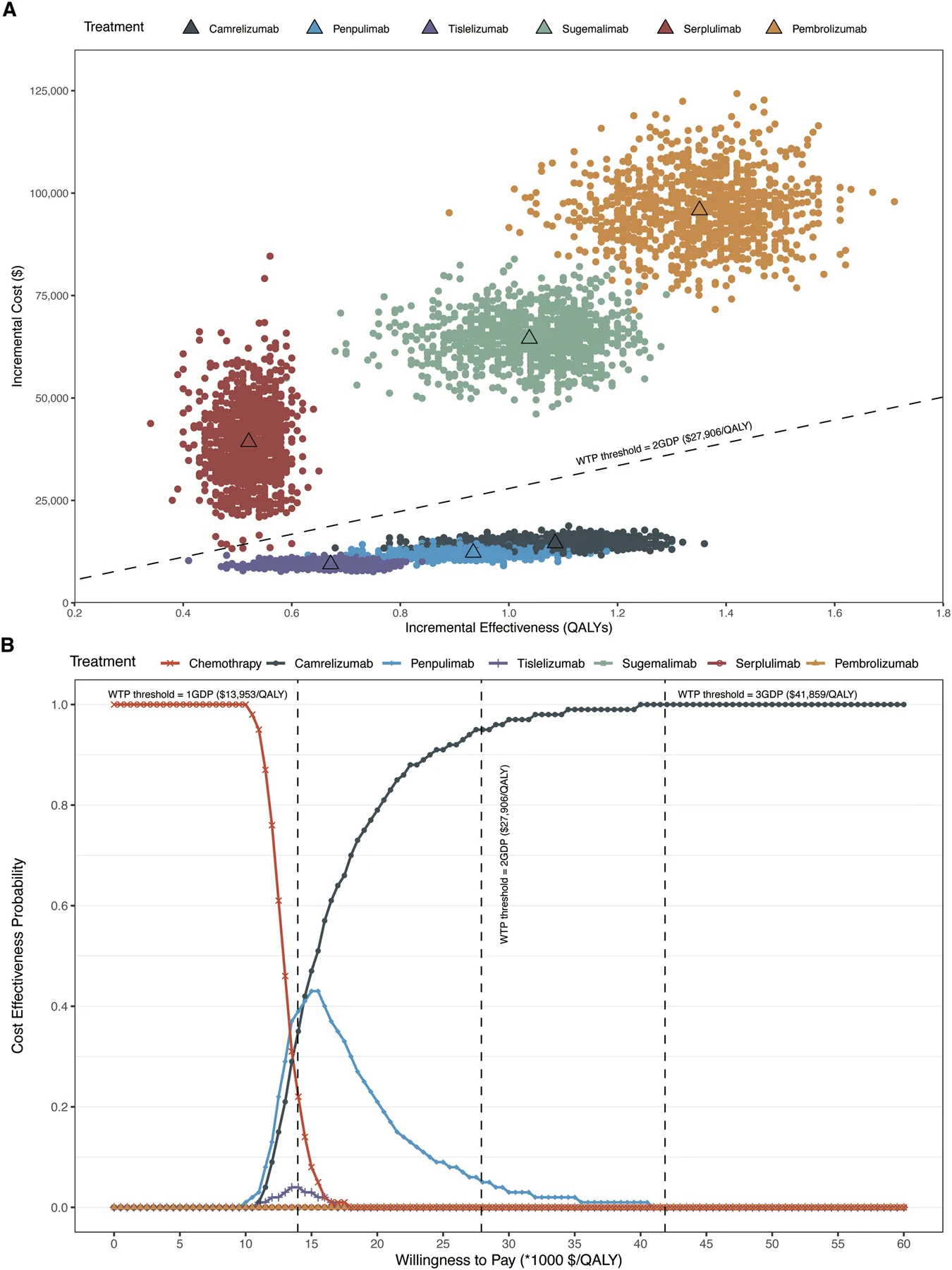
Probabilistic sensitivity analysis of six ICI-plus-chemotherapy regimens compared with chemotherapy alone. **(A)** Incremental cost-effectiveness scatterplots based on 1,000 probabilistic simulations. Each point represents one simulation result for the incremental cost and incremental effectiveness of each regimen relative to chemotherapy. **(B)** Cost-effectiveness acceptability curves for six ICI-based regimens and chemotherapy. The curves show the probability that each treatment is the most cost-effective option across a range of willingness-to-pay thresholds. Abbreviation: PFS = progression-free survival; PD = progressed disease; CT = computed tomography; QALY = quality-adjusted life-year; ICER = incremental cost-effectiveness ratio; WTP = willingness-to-pay.

The CEAC showed that the preferred treatment strategy varied across WTP thresholds (Figure 5B; Supplementary Figure S5). At $13,953/QALY, penpulimab plus chemotherapy and camrelizumab plus chemotherapy had cost-effectiveness probabilities of 39% and 35%, respectively (Figure 5B). However, at the prespecified threshold of $27,906/QALY, camrelizumab plus chemotherapy had the highest probability of being cost-effective (95%), whereas the corresponding probability for penpulimab plus chemotherapy decreased to 5% and remained close to zero for the other competing regimens and chemotherapy alone. At the threshold of $41,859/QALY, camrelizumab plus chemotherapy retained the highest probability of being cost-effective. At higher WTP thresholds (Supplementary Figure S5), however, the probability of pembrolizumab plus chemotherapy being cost-effective increased steadily, while that of camrelizumab plus chemotherapy gradually declined. By contrast, the remaining competing regimens consistently showed low cost-effectiveness probabilities across the evaluated threshold range.

### 3.3 Scenario analyses

Scenario analyses generally supported the base-case cost-effectiveness conclusions (Table 2). In Scenario 1, reductions in ICI acquisition prices progressively lowered the ICERs and increased the INMBs of all six regimens. Penpulimab (Supplementary Figure S6), tislelizumab (Supplementary Figure S7), and camrelizumab (Figure 6) plus chemotherapy remained cost-effective compared with chemotherapy alone across all evaluated price levels. Pembrolizumab (Figure 7), sugemalimab (Supplementary Figure S8), and serplulimab (Supplementary Figure S9) plus chemotherapy remained above the WTP threshold when their prices were set at 90%, 75%, or 50% of the current levels, but became cost-effective compared with chemotherapy alone when their prices were reduced to 25% of the current levels. Shortening the time horizon to 10 years or 8 years did not change the overall cost-effectiveness classification. Penpulimab plus chemotherapy yielded the lowest ICER in both the 10-year and 8-year scenarios ($12,443.36 and $11,786.90 per QALY, respectively), followed by camrelizumab plus chemotherapy ($13,067.83 and $12,728.61 per QALY) and tislelizumab plus chemotherapy ($13,650.20 and $13,280.48 per QALY). In Scenario 4 and 5, tislelizumab, penpulimab, and camrelizumab plus chemotherapy remained cost-effective, whereas sugemalimab, serplulimab, and pembrolizumab plus chemotherapy remained above the WTP threshold.

**FIGURE 6:**
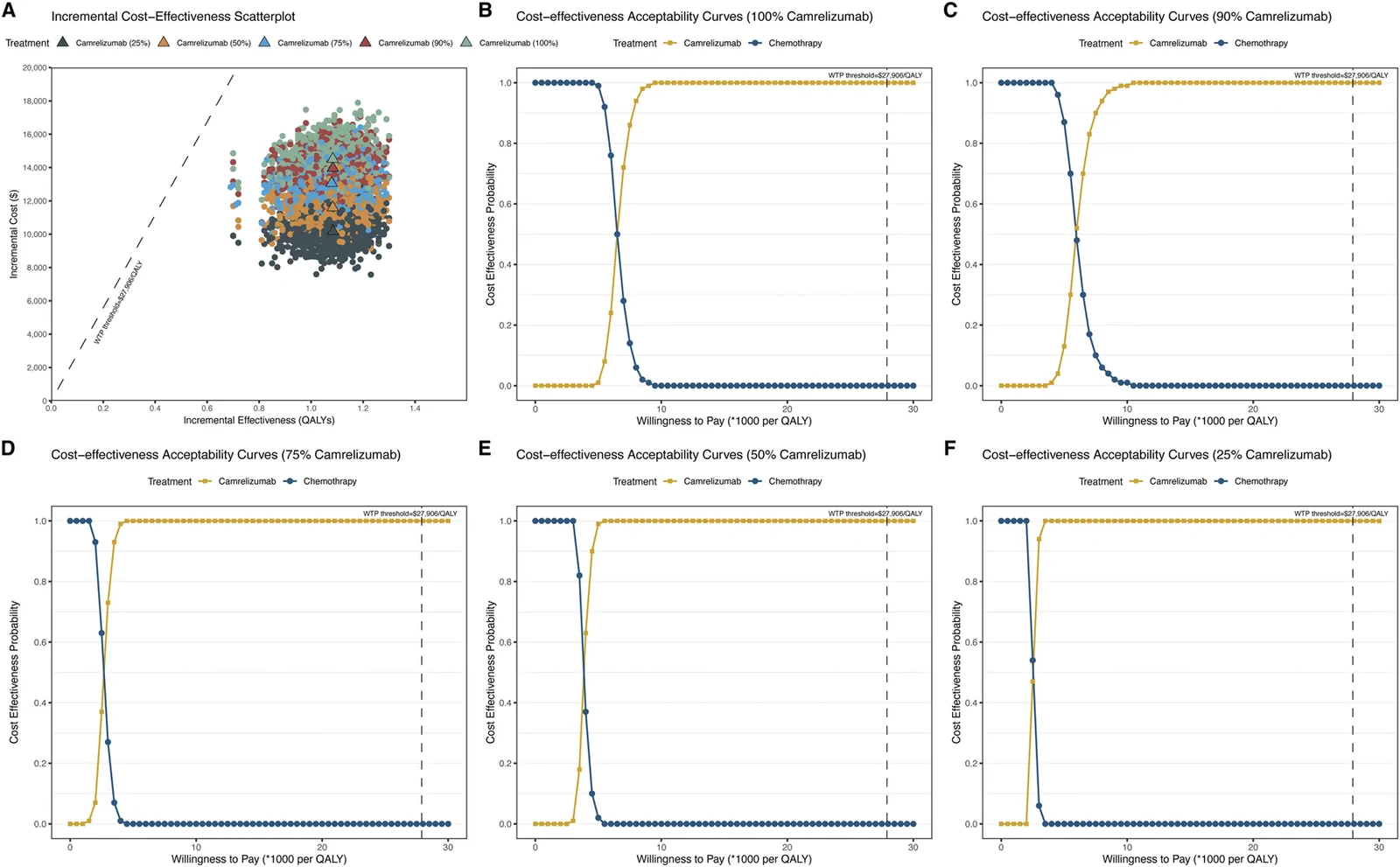
Probabilistic sensitivity analysis **(A)** and cost-effectiveness acceptability curves for camrelizumab plus chemotherapy when the acquisition price of camrelizumab was set at 100% **(B)**, 90% **(C)**, 75% **(D)**, 50% **(E)**, and 25% **(F)** of the current cost. Abbreviation: WTP = willingness-to-pay; QALY = quality-adjusted life-year.

**FIGURE 7:**
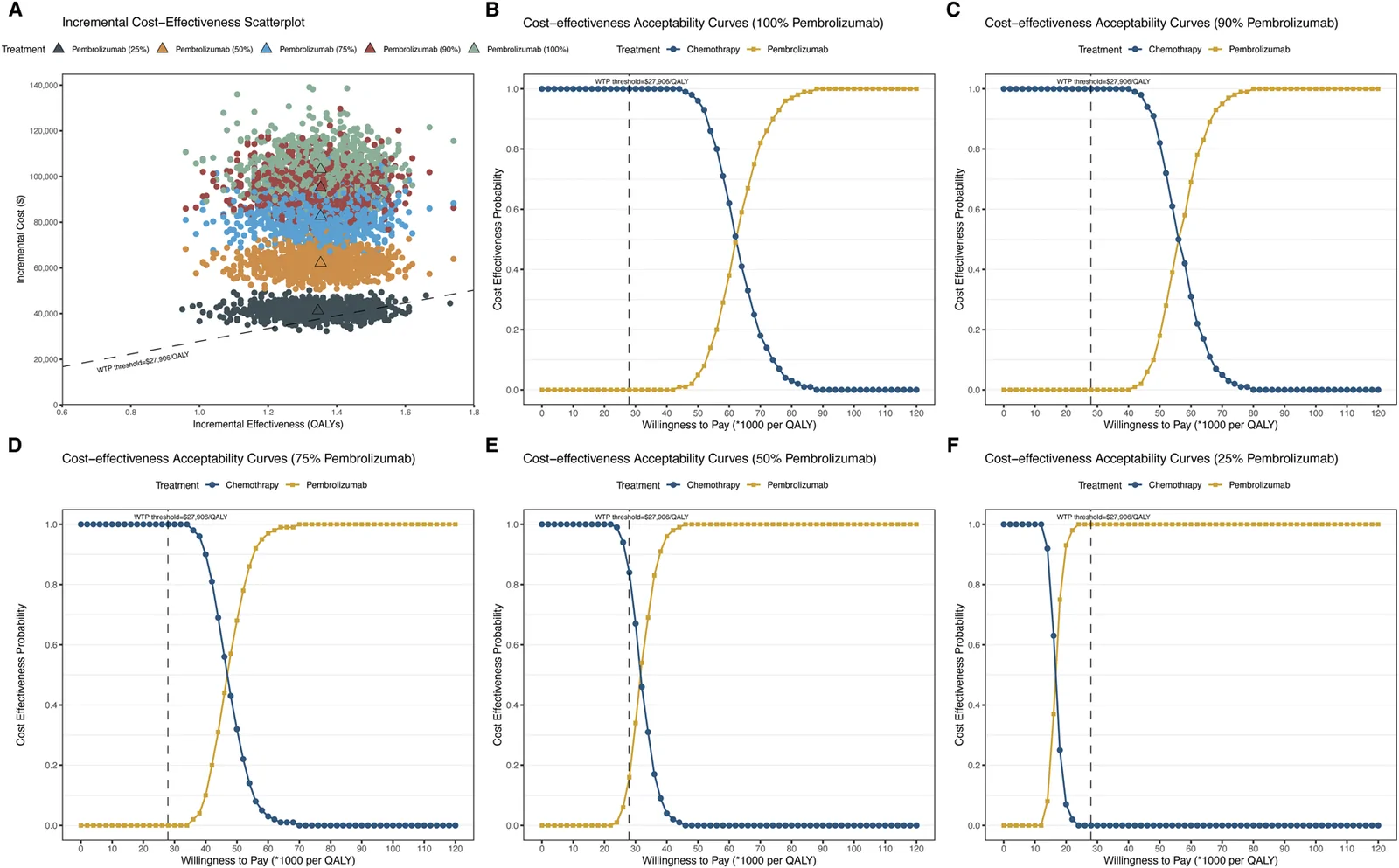
Probabilistic sensitivity analysis **(A)** and cost-effectiveness acceptability curves for pembrolizumab plus chemotherapy when the acquisition price of pembrolizumab was set at 100% **(B)**, 90% **(C)**, 75% **(D)**, 50% **(E)**, and 25% **(F)** of the current cost. Abbreviation: WTP = willingness-to-pay; QALY = quality-adjusted life-year.

## 4 Discussion

Immunochemotherapy is now widely recognized as a safe and effective strategy for the treatment of advanced sq-NSCLC. This recognition has heightened interest among healthcare decision makers, researchers, and clinicians involved in the economic evaluation of treatment alternatives. Selection of the optimal strategy should be guided by a country-specific assessment of relative costs and benefits. In this study, we compared the cost-effectiveness of six ICI-based first-line regimens for advanced sq-NSCLC using a PSM informed by a NMA of phase three evidence from the perspective of the Chinese healthcare-system. All ICI-based strategies improved LYs and QALYs relative to chemotherapy alone. However, these clinical benefits were not matched by uniform economic value. At the prespecified WTP threshold of $27,906 per QALY, camrelizumab plus chemotherapy emerged as the regimen most likely to be cost-effective. By contrast, pembrolizumab plus chemotherapy produced the greatest health gain but was not cost-effective compared with chemotherapy alone at current prices, and sugemalimab plus chemotherapy and serplulimab plus chemotherapy also remained above the threshold in the base-case analysis. These findings underscore oncology’s enduring challenge of balancing innovation-driven survival gains with treatment affordability.

The markedly different economic profile of pembrolizumab plus chemotherapy was primarily driven by the interaction between its favorable modelled efficacy and substantially higher acquisition cost. The OS and PFS HRs derived from the KEYNOTE-407 Chinese cohort were 0.44 and 0.35, respectively, resulting in the highest projected health benefit among the evaluated strategies, with 4.16 LYs and 2.07 QALYs. However, pembrolizumab was priced at $2,507.87 per 100 mg and administered at a fixed dose of 200 mg every 3 weeks, resulting in a substantially higher per-cycle acquisition cost than that of most domestically developed ICIs. In addition, the longer modelled PFS increased treatment exposure and associated medical resource use. Consequently, pembrolizumab plus chemotherapy incurred the highest total cost of $108,217.87 and an ICER of $70,913.04/QALY versus chemotherapy alone. By comparison, penpulimab plus chemotherapy and camrelizumab plus chemotherapy generated slightly smaller health gains but at markedly lower acquisition and total costs. The price-reduction analyses further supported this explanation: the ICER for pembrolizumab plus chemotherapy decreased to $40,229.63/QALY when its price was reduced to 50% of the current level and to $25,027.14/QALY when its price was reduced to 25%. Thus, the high ICER of pembrolizumab plus chemotherapy reflected the inability of its additional health gain to offset its substantially higher acquisition cost at the current price in China, rather than limited clinical benefit. This finding is consistent with previous economic evaluations in China, which have generally shown that pembrolizumab-based regimens are unlikely to be cost-effective unless patient-assistance programs or substantial price reductions are considered (Wan et al., 2025; Courtney et al., 2025; Zhu et al., 2022; Zheng et al., 2023). Likewise, serplulimab plus chemotherapy has been reported as not cost-effective for first-line treatment of extensive-stage small-cell lung cancer in China (Long et al., 2023), and sugemalimab plus CAPOX was not cost-effective for advanced or metastatic gastric cancer from the perspective of Taiwan’s healthcare payer unless a 20%–30% price reduction was assumed (Chueh et al., 2025). By contrast, several domestically developed PD-1/PD-L1 inhibitors, including camrelizumab, and tislelizumab, have demonstrated more favorable economic profiles, largely because of their lower acquisition prices following reimbursement negotiation and market competition (Zou et al., 2025; Zhe et al., 2025; Tang et al., 2024).

The sensitivity and scenario analyses further clarify which factors truly drive the economic conclusions. The model was most sensitive to the utility values assigned to PFS and PD, as well as to ICI acquisition costs. This is expected. In immuno-oncology, health-state utility values determine how effectively survival gains are converted into QALYs, whereas drug prices directly determine whether incremental costs remain within society’s WTP. Notably, although variation in these parameters altered the magnitude of ICERs, it did not change the overall conclusions for camrelizumab, penpulimab, and tislelizumab plus chemotherapy, indicating that the main findings are robust. By contrast, serplulimab plus chemotherapy was more sensitive to body weight, suggesting that weight-based dosing strategies may be more vulnerable to patient heterogeneity in real-world practice. Scenario analyses reinforced the same message. Regimens that were already cost-effective remained favorable across alternative price and time-horizon assumptions, whereas sugemalimab and serplulimab plus chemotherapy became cost-effective only after substantial price reductions. These results indicate that the value of ICIs is fundamentally shaped by price negotiation, rather than determined independently of them (Mahumud et al., 2026).

These findings also have broader clinical and policy relevance for China. Advanced sq-NSCLC continues to impose a substantial disease and economic burden, and first-line chemoimmunotherapy has become an important therapeutic direction in routine care. When efficacy differences are modest or clinically comparable, the decision regarding which immunotherapy regimen should be prioritized for reimbursement, and whether clinicians should incorporate economic evidence into their treatment decisions, remains a critical and unresolved question in oncology practice. In that sense, our study contributes to the evidence base for value-based oncology payment and more precise resource allocation. This perspective is increasingly important because approval or uptake of new anticancer drugs cannot be justified by survival benefit alone. Economic context also matters, particularly when innovative agents are layered onto existing regimens and treatment costs rise sharply. This issue is especially relevant in China, where substantial regional differences in GDP *per capita* translate into marked variation in implicit WTP thresholds. Reported WTP values per QALY range from $80,822 in Beijing and $76,277 in Shanghai to $43,281 in Guangdong, $39,188 in Hubei, $36,650 in Hunan, $22,200 in Guangxi, and $19,127 in Gansu (Li et al., 2024; Li et al., 2022). A regimen such as pembrolizumab plus chemotherapy might become acceptable in more affluent provinces, whereas the same conclusion would not hold in less developed settings. Regional economic heterogeneity in China should therefore be considered when interpreting cost-effectiveness evidence for local implementation.

Clinical selection among these six regimens should not be based on cost-effectiveness alone. Physician preferences were not directly measured in this study and may vary according to guideline recommendations, approved indications, the maturity and magnitude of the survival evidence, PD-L1 expression, ECOG performance status, comorbidities, contraindications to immunotherapy, toxicity profiles, chemotherapy backbones, dosing and monitoring requirements, local formulary availability, reimbursement arrangements, and patient preferences. Within the Chinese healthcare-system perspective, at the current modelled prices and a WTP threshold of $27,906 per QALY, the full incremental analysis identified camrelizumab plus chemotherapy as the preferred strategy after dominated alternatives were excluded, while penpulimab plus chemotherapy also remained on the efficiency frontier. Tislelizumab plus chemotherapy had a favorable ICER compared with chemotherapy alone but was subject to extended dominance in the full incremental comparison. Pembrolizumab plus chemotherapy generated the greatest modelled health benefit but was not cost-effective at the selected threshold, whereas sugemalimab and serplulimab plus chemotherapy also exceeded the threshold at their current prices. These results may inform price negotiations, resource allocation, and shared decision-making when multiple regimens are clinically appropriate. However, they should not be interpreted as a prescriptive ranking for an individual patient because the comparative evidence was indirect and the model did not capture all patient-level heterogeneity or local access constraints.

The transferability of these findings to healthcare systems outside China also requires careful consideration. This analysis was conducted from the perspective of the Chinese healthcare-system and incorporated China-specific drug acquisition prices, and WTP threshold. In other jurisdictions, access to individual ICIs may differ because of variations in regulatory approval, formulary inclusion, procurement and price-negotiation mechanisms, and the capacity to deliver and monitor novel therapies. Differences in administration costs, management of immune-related AEs, and local decision thresholds may also substantially alter total costs and cost-effectiveness rankings, even when relative clinical effects are assumed to be similar. Therefore, the numerical ICERs and the identification of camrelizumab plus chemotherapy as the strategy most likely to be cost-effective should not be directly extrapolated to other countries. Application of this model in other settings would require jurisdiction-specific adaptation of drug prices, treatment pathways, survival assumptions, and cost-effectiveness thresholds.

Several limitations should be acknowledged. First, the relative efficacy estimates were based on indirect comparisons across phase 3 trials rather than head-to-head evidence. The NMA did not formally adjust for cross-trial differences in geographic setting, patient characteristics, histological composition, ECOG performance status, PD-L1 distribution, chemotherapy backbone, follow-up duration, crossover, or subsequent therapy. Because the evidence network contained no closed loops, and each regimen was primarily informed by a single trial, inconsistency and within-comparison heterogeneity could not be estimated. Potential violations of the transitivity assumption may therefore have affected the indirect treatment effects and the resulting QALYs and ICERs (Qi et al., 2026). Second, treatment-specific survival was generated by applying constant HRs to a common chemotherapy baseline curve derived from the GEMSTONE-302 sq-NSCLC subgroup. Although this subgroup was relevant to the Chinese target population and provided relatively mature survival data, the results remained dependent on the representativeness of the selected baseline, the proportional hazards assumption, and the parametric extrapolation models. A scenario analysis using the CameL-Sq chemotherapy arm as an alternative baseline was conducted, but residual structural uncertainty could not be fully excluded. Third, the health-state utility values were obtained from published economic evaluations rather than measured prospectively and consistently across the six pivotal trials. Although these values have been commonly used in advanced sq-NSCLC CEAs, they may not fully reflect the health-related quality of life of contemporary Chinese patients with advanced sq-NSCLC receiving different ICI-based regimens. Nevertheless, the influence of utility uncertainty was examined in the deterministic and probabilistic sensitivity analyses. Fourth, AE modelling was simplified by including only grade 3 or higher events with an incidence exceeding 5% and applying the associated costs and disutilities during the first model cycle. Although this approach captured the principal severe toxicities reported in the pivotal trials, the model may not fully capture less frequent, delayed-onset, recurrent, persistent, or prolonged immune-related AEs that emerge beyond the initial treatment cycle and require extended monitoring or management (Zhao et al., 2025). Fifth, post-progression management was simplified by assuming that all patients received best supportive care after disease progression, with the corresponding cost applied during each cycle spent in the PD state. Differences in subsequent anticancer treatment, including regimen-specific chemotherapy, immunotherapy, treatment switching, and crossover, were not explicitly modelled because comparable treatment-level data were not consistently available across the included trials. Consequently, the model may not fully reflect between-trial differences in post-progression treatment patterns, costs, and their potential effects on OS, particularly because crossover and subsequent immunotherapy were common in several control groups. Sixth, the cost-effectiveness results were specific to China. Drug prices and medical costs may vary among countries and may change following price negotiations. The WTP threshold used in the analysis was also specific to China. Consequently, the numerical ICERs and cost-effectiveness rankings should not be directly generalized to other jurisdictions without recalculation using local cost inputs and WTP thresholds. Finally, the findings reflect the available clinical evidence and drug prices at the time of the analysis. Longer-term trial data, head-to-head comparisons, real-world evidence, and future changes in drug prices may alter comparative effectiveness and cost-effectiveness estimates. Periodic updating of the model will therefore be required.

## 5 Conclusion

In summary, ICI–chemotherapy combinations improved health outcomes compared with chemotherapy alone for patients with advanced sq-NSCLC, although their economic value varied substantially at current prices in China. Under the assumptions of the present model and at a WTP threshold of $27,906 per QALY, camrelizumab, penpulimab, and tislelizumab plus chemotherapy appeared cost-effective in pairwise comparisons with chemotherapy alone. In the full incremental analysis, after strictly and extendedly dominated options were excluded, camrelizumab plus chemotherapy was the preferred strategy at the selected WTP threshold. Compared with chemotherapy alone, higher-cost regimens, particularly pembrolizumab, sugemalimab, and serplulimab plus chemotherapy, were unlikely to be cost-effective without substantial price reductions or higher decision thresholds. These findings should be interpreted cautiously because they depend on indirect treatment comparisons across separate trials, the selected chemotherapy baseline, the drug-price inputs, the proportional-hazards and survival-extrapolation assumptions, and the simplified modelling of post-progression treatment. The estimated ICERs and treatment rankings may change with alternative baseline survival data, updated prices, different extrapolation approaches, more detailed subsequent-treatment pathways, head-to-head evidence, or longer-term clinical follow-up. Accordingly, these results may inform, rather than determine, value-based treatment selection and healthcare resource allocation for advanced sq-NSCLC in China.

## Data Availability

The original contributions presented in the study are included in the article/Supplementary Material, further inquiries can be directed to the corresponding author.
