# Supplementary material for: Economic evaluation of six immune checkpoint inhibitor-based first-line therapies for advanced squamous non-small-cell lung cancer: a cost-effectiveness analysis based on network meta-analysis
Source: Front Pharmacol. 2026 Oct 13;17:1841871. doi: 10.3389/fphar.2026.1841871 (PMC13710048; doi:10.3389/fphar.2026.1841871)
Supplement: Supplementary file 1 [file DataSheet1.pdf]

Supplementary File:

Table S1. Characteristics and key clinical outcomes of the pivotal phase 3 trials for squamous NSCLC included in the analysis. 3
Table S2. Survival estimates entered into the network meta-analysis and assessment of trial comparability. 5
Table S3. CHEERS 2022 Checklist. 10
Table S4. AIC and BIC values for Kaplan-Meier curve fitting distribution in chemotherapy group from the squamous NSCLC subgroup in GEMSTONE-302. 13
Table S5. Study characteristics and hazard ratio data for overall survival and progression-free survival included in the network meta-analysis. 14
Table S6. Incidence rates of treatment-related adverse events. 15
Table S7. Breakdown of costs and health outcomes by treatment strategy. 17
Table S8. Full incremental cost-effectiveness analysis of all treatment strategies. 19
Figure S1. Kaplan-Meier curves for overall (A) and progression-free (B) survival from the squamous NSCLC subgroup in GEMSTONE-302 study. 20
Figure S2. Reconstructed Kaplan–Meier data and fitted parametric survival curves for overall survival (A) and progression-free survival (B) in the chemotherapy arm of the GEMSTONE-302 squamous NSCLC subgroup. 21
Figure S3. Network meta-analysis of eligible comparisons for clinical efficacy. Each node represents a treatment regimen, with the node size proportional to the total number of randomized patients receiving that treatment. Lines represent direct head-to-head comparisons; line thickness corresponds to the number of trials (all = 1 in this network). The network exhibits a predominantly star-shaped structure with the platinum-based chemotherapy serving as the common comparator. 22
Figure S4. Forest plots of the treatment effects of six first-line ICI-plus-chemotherapy regimens versus chemotherapy alone. (A) Hazard ratios for overall survival. (B) Hazard ratios for progression-free survival. 23
Figure S5. Cost-effectiveness acceptability curves for six ICI-based regimens and chemotherapy. 24
Figure S6. Probabilistic sensitivity analysis (A) and cost-effectiveness acceptability curves for penpulimab plus chemotherapy when the acquisition price of penpulimab was set at 100% (B), 90% (C), 75% (D), 50% (E), and 25% (F) of the current cost. 25
Figure S7. Probabilistic sensitivity analysis (A) and cost-effectiveness acceptability curves for tislelizumab plus chemotherapy when the acquisition price of tislelizumab was set at 100% (B), 90% (C), 75% (D), 50% (E), and 25% (F) of the current cost. 26
Figure S8. Probabilistic sensitivity analysis (A) and cost-effectiveness acceptability curves for sugemalimab plus chemotherapy when the acquisition price of sugemalimab was set at 100% (B), 90% (C), 75% (D), 50% (E), and 25% (F) of the current cost. 27

Figure S9. Probabilistic sensitivity analysis (A) and cost-effectiveness acceptability curves for serplulimab plus chemotherapy when the acquisition price of serplulimab was set at 100% (B), 90% (C), 75% (D), 50% (E), and 25% (F) of the current cost........ 28

Table S1. Characteristics and key clinical outcomes of the pivotal phase 3 trials for squamous NSCLC included in the analysis.

Trial	Treatment arms and sample size	Chemotherapy backbone	Median follow-up	Median PFS, months; HR (95% CI)	Median OS, months; HR (95% CI)	Key safety findings
GEMSTONE-302	The squamous subgroup included 129 and 63 patients, respectively	For squamous NSCLC: carboplatin AUC 5 plus paclitaxel 175 mg/m² every 3 weeks for up to 4 cycles.	43.5 months versus 43.0 months	For squamous NSCLC: 8.3 versus 4.8; 0.34 (0.24–0.48)	For squamous NSCLC: 23.6 versus 12.2; 0.61 (0.43–0.87)	The main severe toxicities were neutropenia (33%), leukopenia (17%), anemia (11%), and thrombocytopenia (9%). No unexpected immune-related safety signals were observed.
RATIONALE-307	Arm A: tislelizumab 200 mg plus paclitaxel–carboplatin, n=120; Arm C: paclitaxel–carboplatin, n=121	Arm A and Arm C: paclitaxel 175 mg/m² plus carboplatin AUC 5. 4–6 cycles	16.7 months	Arm A versus C: 7.7 versus 5.5; 0.45 (0.33–0.62).	Arm A versus C: 22.8 versus 20.2; 0.69 (0.50–0.95).	The main severe toxicities were neutropenia (33%), leukopenia (16%), anemia (10%), and thrombocytopenia (7%). No unexpected immune-related safety signals were observed.
AK105-302	Penpulimab 200 mg plus chemotherapy, n=175; placebo plus chemotherapy, n=175	Paclitaxel 175 mg/m² plus carboplatin AUC 5 every 3 weeks for 4 cycles, followed by penpulimab or placebo maintenance	24.7 months	7.6 versus 4.2; 0.43 (0.33–0.56)	NR versus 20.2; 0.55 (0.40–0.75)	The main severe toxicities were neutropenia (45%) and leukopenia (20%). No unexpected immune-related safety signals were observed.
ASTRUM-004	Serplulimab 4.5 mg/kg plus chemotherapy, n=358; placebo plus chemotherapy, n=179	Nab-paclitaxel 100 mg/m² on days 1, 8, and 15 plus carboplatin AUC 5 or 6 every 3 weeks for 4–6 cycles	PFS update: 16.9 months; final OS analysis: 31.1 months	8.3 versus 5.7; 0.53 (0.42–0.67)	22.7 versus 18.2; 0.73 (0.58–0.93)	The main severe toxicities were neutropenia (5%), leukopenia (10%), anemia (12%), and thrombocytopenia (7%). No unexpected immune-related safety signals were observed.

Trial	Treatment arms and sample size	Chemotherapy backbone	Median follow-up	Median PFS, months; HR (95% CI)	Median OS, months; HR (95% CI)	Key safety findings
CameL-Sq	Camrelizumab 200 mg plus chemotherapy, n=193; placebo plus chemotherapy, n=196	Paclitaxel 175 mg/m² plus carboplatin AUC 5 every 3 weeks for 4–6 cycles, followed by camrelizumab or placebo maintenance	13.5 months versus 11.6 months	8.5 versus 4.9; 0.37 (0.29–0.47)	NR versus 14.5; 0.55 (0.40–0.75)	The main severe toxicities were neutropenia (55%), leukopenia (30%), anemia (10%), and thrombocytopenia (7%). No unexpected immune-related safety signals were observed.
KEYNOTE-407 China cohort	Pembrolizumab 200 mg plus chemotherapy, n=65; placebo plus chemotherapy, n=60	Carboplatin AUC 6 plus paclitaxel 200 mg/m² every 3 weeks for 4 cycles. All patients in the Chinese cohort received paclitaxel rather than nab-paclitaxel	28.1 months	8.3 versus 4.2; 0.35 (0.24–0.52)	30.1 versus 12.7; 0.44 (0.28–0.70)	The main severe toxicities were neutropenia (62%), leukopenia (35%), anemia (5%), and thrombocytopenia (8%). No unexpected immune-related safety signals were observed.

Abbreviations: NSCLC, non-small-cell lung cancer; AE, adverse event; AUC, area under the concentration–time curve; CI, confidence interval; ECOG PS, Eastern Cooperative Oncology Group performance status; HR, hazard ratio; NE, not estimable; NR, not reached; OS, overall survival; PD-L1, programmed death ligand 1; PFS, progression-free survival; TEAE, treatment-emergent adverse event.

Table S2. Survival estimates entered into the network meta-analysis and assessment of trial comparability.

Trial and comparison	Study population and disease stage	Geographic setting and design	PD-L1 eligibility	ECOG PS	Analysis population used for efficacy estimate	PFS HR (95% CI) used in NMA	OS HR (95% CI) used in NMA	Maturity and crossover-adjusted evidence	Application in model	Protocol-permitted crossover from control arm	Subsequent treatment in the control arm
GEMSTONE-302: Sugemalimab plus chemotherapy versus chemotherapy	Previously untreated stage IV squamous NSCLC without sensitizing EGFR mutations or ALK, ROS1, or RET fusions	China; 35 centers; randomized, double-blind, placebo-controlled phase 3 trial	No PD-L1 restriction; analyses were conducted across PD-L1 subgroups	0–1	The squamous subgroup of GEMSTONE-302	0.34 (0.24–0.48)	0.61 (0.43–0.87)	Four-year updated analysis; OS relatively mature. No crossover-adjusted primary estimate was used	The chemotherapy-arm OS and PFS curves from the squamous NSCLC subgroup were reconstructed and extrapolated as the common baseline survival curves. The squamous subgroup HRs for OS and PFS were used as the comparative efficacy inputs	Permitted; 46/159 patients (28.9%) crossed over to open-label sugemalimab	110/159 patients (69.2%) received subsequent anticancer therapy; 71/159 (44.7%) received subsequent immunotherapy

Trial and comparison	Study population and disease stage	Geographic setting and design	PD-L1 eligibility	ECOG PS	Analysis population used for efficacy estimate	PFS HR (95% CI) used in NMA	OS HR (95% CI) used in NMA	Maturity and crossover-adjusted evidence	Application in model	Protocol-permitted crossover from control arm	Subsequent treatment in the control arm
									for sugemalimab.		
RATIONALE-307: Tislelizumab plus paclitaxel–carboplatin versus paclitaxel–carboplatin	Previously untreated, unresectable stage IIIB or metastatic stage IV squamous NSCLC	China; multicentre, randomized, open-label phase 3 trial	No restriction; stratified as <1%, 1–49%, and ≥50% tumor-cell expression	0–1	RATIONALE-307 Arm A versus Arm C	0.45 (0.33–0.62)	0.69 (0.50–0.95)	Updated unadjusted OS HR: 0.69; crossover-adjusted two-stage OS HR: 0.57. The unadjusted trial estimate was retained for the primary NMA	HRs applied to the common chemotherapy baseline curves	Permitted; 71/121 patients (58.7%) had crossed over to tislelizumab by the updated analysis	77/121 patients (63.6%) received subsequent immunotherapy
AK105-302: Penpulimab plus chemotherapy versus chemotherapy	Previously untreated stage IIIB/IIIC or IV squamous NSCLC unsuitable for curative surgery or chemoradiotherapy	China; 74 hospitals; randomized, double-blind, placebo-	No restriction; stratified as <1%, 1–49%, and ≥50% TPS	0–1	ITT population of AK105-302	0.43 (0.33–0.56)	0.55 (0.40–0.75)	Median OS was not reached versus 20.2 months; crossover-adjusted	HRs applied to the common chemotherapy baseline curves	Permitted; 102/175 patients (58.3%) crossed over to penpulimab	Other subsequent treatments were not reported consistently in the primary publication

Trial and comparison	Study population and disease stage	Geographic setting and design	PD-L1 eligibility	ECOG PS	Analysis population used for efficacy estimate	PFS HR (95% CI) used in NMA	OS HR (95% CI) used in NMA	Maturity and crossover-adjusted evidence	Application in model	Protocol-permitted crossover from control arm	Subsequent treatment in the control arm
		controlled phase 3 trial						sensitivity-analysis OS HR was 0.45 (0.29–0.70)			
ASTRUM-004: Serplulimab plus chemotherapy versus chemotherapy	Previously untreated stage IIIB/IIIC or IV squamous NSCLC without targetable genomic alterations and unsuitable for curative treatment	Global; 85 sites across six countries; randomized, double-blind, placebo-controlled phase 3 trial; approximately 67% Asian and 33% White participants	No restriction; stratified as <1%, 1–49%, and ≥50% TPS	0–1	ITT population of ASTRUM-004	0.53 (0.42–0.67)	0.73 (0.58–0.93)	Final OS analysis; crossover-adjusted two-stage OS HR was 0.49 (0.37–0.64)	HRs applied to the common chemotherapy baseline curves	Permitted; 97/179 patients (54.2%) crossed over to serplulimab	Subsequent therapies other than protocol-specified crossover were not reported consistently
CameL-Sq: Camrelizumab plus	Previously untreated stage IIIB–IV squamous NSCLC	China; 53 centers; randomized,	No restriction; approximately	0–1	ITT population of CameL-Sq	0.37 (0.29–0.47)	0.55 (0.40–0.75)	Median OS was not reached versus 14.5	HRs applied to the common	Permitted; approximately 47% crossed	Approximately 53% of control-arm patients

Trial and comparison	Study population and disease stage	Geographic setting and design	PD-L1 eligibility	ECOG PS	Analysis population used for efficacy estimate	PFS HR (95% CI) used in NMA	OS HR (95% CI) used in NMA	Maturity and crossover-adjusted evidence	Application in model	Protocol-permitted crossover from control arm	Subsequent treatment in the control arm
chemotherapy versus chemotherapy		double-blind, placebo-controlled phase 3 trial	51% had PD-L1 TPS <1%					months; RPSFT-adjusted OS HR was 0.50 (0.35–0.72)	chemotherapy baseline curves	over to camrelizumab after progression	received a subsequent PD-1/PD-L1 inhibitor
KEYNOTE-407: Pembrolizumab plus chemotherapy versus chemotherapy	Previously untreated stage IV squamous NSCLC	Mainland China; patients pooled from the global study and China extension; randomized, double-blind, placebo-controlled phase 3 design	No restriction	0–1	Chinese cohort of KEYNOTE-407	0.35 (0.24–0.52)	0.44 (0.28–0.70)	Median OS was 30.1 versus 12.7 months. Effective crossover to pembrolizumab was 63.3%, and 65.0% received subsequent anti-PD-(L)1 therapy	HRs applied to the common chemotherapy baseline curves	Permitted; 38/60 patients (63.3%) crossed over to pembrolizumab	46/60 patients (76.7%) received subsequent therapy; 39/60 (65.0%) received a subsequent anti-PD-(L)1 therapy

Note: The trial-specific subsequent-treatment patterns shown in this table were used to assess clinical comparability and were not directly reproduced in the economic model. Post-progression management in the model followed the prespecified common assumption described in the Methods. Abbreviations: AUC, area under the concentration–time curve; CI, confidence interval; ECOG PS, Eastern Cooperative Oncology Group performance status; HR, hazard ratio; ICI, immune checkpoint inhibitor; ITT, intention-to-treat; NMA, network meta-analysis; NR, not reached

or not reported; NSCLC, non-small-cell lung cancer; OS, overall survival; PD-L1, programmed death ligand 1; PFS, progression-free survival; RPSFT, rank-preserving structural failure time; TPS, tumor proportion score.

Table S3. CHEERS 2022 Checklist.

Topic	No.	Item	Reported
**Title**	1	Identify the study as an economic evaluation and specify the interventions being compared.	Yes
**Abstract**	2	Provide a structured summary that highlights context, key methods, results, and alternative analyses.	Yes
**Introduction**			
**Background and objectives**	3	Give the context for the study, the study question, and its practical relevance for decision making in policy or practice.	Yes
**Methods**			
**Health economic analysis plan**	4	Indicate whether a health economic analysis plan was developed and where available.	Not applicable
**Study population**	5	Describe characteristics of the study population (such as age range, demographics, socioeconomic, or clinical characteristics).	Yes
**Setting and location**	6	Provide relevant contextual information that may influence findings.	Yes
**Comparators**	7	Describe the interventions or strategies being compared and why chosen.	Yes
**Perspective**	8	State the perspective(s) adopted by the study and why chosen.	Yes
**Time horizon**	9	State the time horizon for the study and why appropriate.	Yes
**Discount rate**	10	Report the discount rate(s) and reason chosen.	Yes
**Selection of outcomes**	11	Describe what outcomes were used as the measure(s) of benefit(s) and harm(s).	Yes
**Measurement of outcomes**	12	Describe how outcomes used to capture benefit(s) and harm(s) were measured.	Yes
**Valuation of outcomes**	13	Describe the population and methods used to measure and value outcomes.	Yes
**Measurement and valuation of resources and costs**	14	Describe how costs were valued.	Yes
**Currency, price date, and conversion**	15	Report the dates of the estimated resource quantities and unit costs, plus the currency and year of conversion.	Yes

Topic	No.	Item	Reported
**Rationale and description of model**	16	If modelling is used, describe in detail and why used. Report if the model is publicly available and where it can be accessed.	Yes
**Analytics and assumptions**	17	Describe any methods for analysing or statistically transforming data, any extrapolation methods, and approaches for validating any model used.	Not applicable
**Characterising heterogeneity**	18	Describe any methods used for estimating how the results of the study vary for subgroups.	Not applicable
**Characterising distributional effects**	19	Describe how impacts are distributed across different individuals or adjustments made to reflect priority populations.	Not applicable
**Characterising uncertainty**	20	Describe methods to characterise any sources of uncertainty in the analysis.	Yes
**Approach to engagement with patients and others affected by the study**	21	Describe any approaches to engage patients or service recipients, the general public, communities, or stakeholders (such as clinicians or payers) in the design of the study.	Not applicable
**Results**			
**Study parameters**	22	Report all analytic inputs (such as values, ranges, references) including uncertainty or distributional assumptions.	Yes
**Summary of main results**	23	Report the mean values for the main categories of costs and outcomes of interest and summarise them in the most appropriate overall measure.	Yes
**Effect of uncertainty**	24	Describe how uncertainty about analytic judgments, inputs, or projections affect findings. Report the effect of choice of discount rate and time horizon, if applicable.	Yes
**Effect of engagement with patients and others affected by the study**	25	Report on any difference patient/service recipient, general public, community, or stakeholder involvement made to the approach or findings of the study	Not applicable
**Discussion**			
**Study findings, limitations, generalisability, and current knowledge**	26	Report key findings, limitations, ethical or equity considerations not captured, and how these could affect patients, policy, or practice.	Yes
**Other relevant information**			

Topic	No.	Item	Reported
**Source of funding**	27	Describe how the study was funded and any role of the funder in the identification, design, conduct, and reporting of the analysis	Yes
**Conflicts of interest**	28	Report authors' conflicts of interest according to journal or International Committee of Medical Journal Editors requirements.	Yes

Table S4. AIC and BIC values for Kaplan-Meier curve fitting distribution in chemotherapy group from the squamous NSCLC subgroup in GEMSTONE-302.

**Parameters**	**Exponential**	**Weibull**	**Gamma**	**Log-logistic**	**Log-normal**	**Gompertz**	**Gen gamma**
**PFS**							
AIC	312.6846	298.4269	294.884	**291.3288**	297.2616	309.6533	296.2565
BIC	314.8277	302.7132	299.1702	**295.6151**	301.5479	313.9396	302.686
**OS**							
AIC	401.5526	403.4799	403.1148	**398.1654**	399.5497	402.8059	401.2695
BIC	403.6957	407.7662	407.401	**402.4516**	403.836	407.0922	407.6989

Abbreviation: AIC = Akaike information criterion. BIC = Bayesian information criterion. PFS = progression-free survival. OS = overall survival.

Table S5. Study characteristics and hazard ratio data for overall survival and progression-free survival included in the network meta-analysis.

Study	NCT number	treat1	treat2	n1	n2	OS					PFS				
						hr	lower	upper	logHR	Se(logHR)	hr	lower	upper	logHR	Se(logHR)
ASTRUM-004	NCT04033354	Serplulimab plus chemotherapy	Chemotherapy alone	358	179	0.73	0.58	0.93	−0.3147	0.1204	0.53	0.42	0.67	−0.6349	0.1191
GEMSTONE-302	NCT03789604	Sugemalimab plus chemotherapy	Chemotherapy alone	129	63	0.61	0.43	0.87	−0.4943	0.1798	0.34	0.24	0.48	−1.0788	0.1768
AK105-302	NCT03866993	Penpulimab plus chemotherapy	Chemotherapy alone	175	175	0.55	0.4	0.75	−0.5978	0.1604	0.43	0.33	0.56	−0.8440	0.1349
CameL-Sq	NCT03668496	Camrelizumab plus chemotherapy	Chemotherapy alone	193	196	0.55	0.4	0.75	−0.5978	0.1604	0.37	0.29	0.47	−0.9943	0.1232
KEYNOTE-407	NCT02775435	Pembrolizumab plus chemotherapy	Chemotherapy alone	65	60	0.44	0.28	0.7	−0.8210	0.2337	0.35	0.24	0.52	−1.0498	0.1972
RATIONALE-307	NCT03594747	Tislelizumab plus chemotherapy	Chemotherapy alone	120	121	0.69	0.5	0.95	−0.3711	0.1637	0.45	0.33	0.62	−0.7985	0.1609

Table S6. Incidence rates of treatment-related adverse events.

Incidence of AEs in various regimens	Baseline value	Range		Distribution	Study
		Minimum	Maximum		
**Chemotherapy alone**					
Leukopenia	0.17	0.14	0.20	Beta	GEMSTONE-302
Neutropenia	0.33	0.26	0.39	Beta	GEMSTONE-302
Anemia	0.11	0.09	0.14	Beta	GEMSTONE-302
Thrombocytopenia	0.09	0.08	0.11	Beta	GEMSTONE-302
**Camrelizumab plus chemotherapy**					
Anemia	0.10	0.08	0.12	Beta	CameL-Sq
Leukopenia	0.30	0.24	0.36	Beta	CameL-Sq
Neutropenia	0.55	0.44	0.67	Beta	CameL-Sq
Thrombocytopenia	0.07	0.05	0.08	Beta	CameL-Sq
**Pembrolizumab plus chemotherapy**					
Anemia	0.05	0.04	0.06	Beta	KEYNOTE-407
Leukopenia	0.35	0.28	0.42	Beta	KEYNOTE-407
Neutropenia	0.62	0.49	0.74	Beta	KEYNOTE-407
Thrombocytopenia	0.08	0.06	0.09	Beta	KEYNOTE-407
**Penpulimab plus chemotherapy**					
Leukopenia	0.20	0.16	0.24	Beta	AK105-302

Incidence of AEs in various regimens	Baseline value	Range		Distribution	Study
		Minimum	Maximum		
Neutropenia	0.45	0.36	0.53	Beta	AK105-302
**Serplulimab plus chemotherapy**					
Anemia	0.12	0.10	0.14	Beta	ASTRUM-004
Leukopenia	0.10	0.08	0.12	Beta	ASTRUM-004
Neutropenia	0.05	0.04	0.06	Beta	ASTRUM-004
Thrombocytopenia	0.07	0.05	0.08	Beta	ASTRUM-004
**Sugemalimab plus chemotherapy**					
Anemia	0.14	0.11	0.17	Beta	GEMSTONE-302
Leukopenia	0.15	0.12	0.18	Beta	GEMSTONE-302
Neutropenia	0.33	0.26	0.39	Beta	GEMSTONE-302
Thrombocytopenia	0.11	0.09	0.13	Beta	GEMSTONE-302
**Tislelizumab plus chemotherapy**					
Anemia	0.10	0.08	0.12	Beta	RATIONALE-307
Leukopenia	0.16	0.13	0.19	Beta	RATIONALE-307
Neutropenia	0.33	0.27	0.40	Beta	RATIONALE-307
Thrombocytopenia	0.07	0.05	0.08	Beta	RATIONALE-307
Pneumonitis	0.05	0.04	0.06	Beta	RATIONALE-307

Table S7. Breakdown of costs and health outcomes by treatment strategy.

Treatment	Chemotherapy alone	Sugemalimab plus chemotherapy	Tislelizumab plus chemotherapy	Penpulimab plus chemotherapy	Serplulimab plus chemotherapy	Camrelizumab plus chemotherapy	Pembrolizumab plus chemotherapy
Total cost/$	12,227.4720	76,492.0778	21,661.2623	24,460.8488	46,852.5772	26,750.8666	108,217.8700
Adverse-event management costs, $	220.2029	244.5273	259.2126	132.8477	137.7485	220.1084	240.1406
End-of-life care costs, $	2,277.2205	2,067.3915	2,140.4264	1,993.3000	2,167.1346	2,048.6156	1,818.9778
Drug acquisition costs, $	8,468.7468	71,758.8514	17,198.7028	19,895.8443	42,664.3489	21,921.5650	103,211.4284
Follow-up and disease-management costs, $	1,261.3018	2,421.3076	2,062.9205	2,438.8568	1,883.3452	2,560.5775	2,947.3232
Costs accrued in the progression-free state, $	2,663.3294	65,236.1291	9,258.7012	8,030.3061	34,002.0546	12,025.4202	89,670.6489
Costs accrued in the progressed disease state, $	7,286.9222	9,188.5572	10,262.1347	14,437.2427	10,683.3879	12,676.8307	16,728.2432
Total LYs	1.5824	3.0874	2.7136	3.4170	2.5510	3.4580	4.1593
Total QALYs	0.7134	1.7523	1.3861	1.6498	1.2351	1.8003	2.0670
QALYs accrued in the progression-free state	0.3581	1.3090	0.8890	0.9497	0.7159	1.1880	1.2589

QALYs accrued in the progressed-disease state	0.3593	0.4479	0.5008	0.7028	0.5218	0.6170	0.8127
Adverse-event-related disutility, QALYs	0.0041	0.0045	0.0037	0.0027	0.0026	0.0047	0.0046

Abbreviation: AE = adverse event; LY = life-year; QALY = quality-adjusted life-year.

Table S8. Full incremental cost-effectiveness analysis of all treatment strategies.

Treatment strategy	Total cost, $	Total LYs	Total QALYs	Comparator on efficiency frontier	Incremental cost, $	Incremental QALYs	Sequential ICER, $/QALY	Economic status
Chemotherapy alone	12,227.47	1.5824	0.7134	Reference	NA	NA	NA	On efficiency frontier
Tislelizumab plus chemotherapy	21,661.26	2.7136	1.3861	NA	NA	NA	NA	Extendedly dominated
Penpulimab plus chemotherapy	24,460.85	3.4170	1.6498	Chemotherapy	12,233.38	0.9371	13,054.86	On efficiency frontier
Camrelizumab plus chemotherapy	26,750.87	3.4580	1.8003	Penpulimab plus chemotherapy	2,290.02	0.1499	15,278.56	On efficiency frontier
Serplulimab plus chemotherapy	46,852.58	2.5510	1.2351	NA	NA	NA	NA	Strictly dominated
Sugemalimab plus chemotherapy	76,492.08	3.0874	1.7523	NA	NA	NA	NA	Strictly dominated
Pembrolizumab plus chemotherapy	108,217.87	4.1593	2.0670	Camrelizumab plus chemotherapy	81,467.00	0.2667	305,489.63	On efficiency frontier; ICER above WTP threshold

Note: Treatment strategies were ordered by increasing total cost. Strict dominance occurred when a strategy was more costly and less effective than another strategy. Extended dominance occurred when a combination of more and less effective strategies provided additional QALYs at a lower incremental cost per QALY. Incremental costs, incremental QALYs, and sequential ICERs were calculated only for strategies remaining on the cost-effectiveness efficiency frontier using unrounded model outputs. At the WTP threshold of $27,906 per QALY, camrelizumab plus chemotherapy was the preferred strategy. Abbreviations: ICER = incremental cost-effectiveness ratio; LY = life-year; QALY = quality-adjusted life-year; WTP = willingness-to-pay.

Figure S1. Kaplan-Meier curves for overall (**A**) and progression-free (**B**) survival from the squamous NSCLC subgroup in GEMSTONE-302 study.

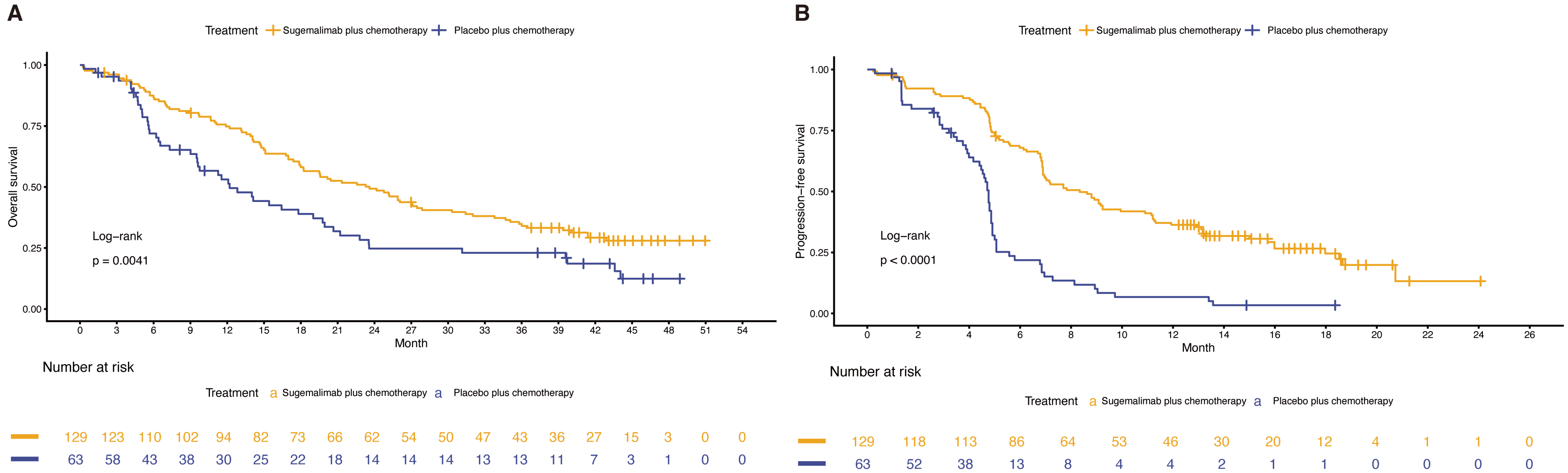

Figure S2. Reconstructed Kaplan–Meier data and fitted parametric survival curves for overall survival (**A**) and progression-free survival (**B**) in the chemotherapy arm of the GEMSTONE-302 squamous NSCLC subgroup.

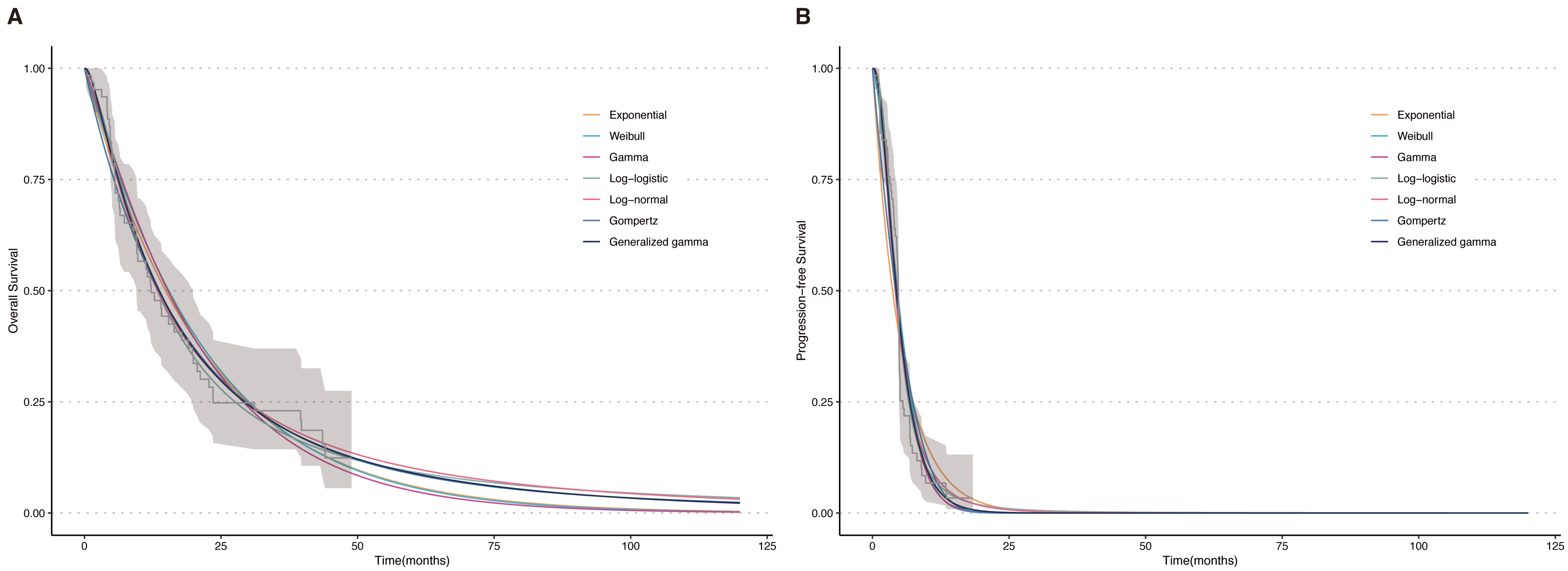

Figure S3. Network meta-analysis of eligible comparisons for clinical efficacy. Each node represents a treatment regimen, with the node size proportional to the total number of randomized patients receiving that treatment. Lines represent direct head-to-head comparisons; line thickness corresponds to the number of trials (all = 1 in this network). The network exhibits a predominantly star-shaped structure with the platinum-based chemotherapy serving as the common comparator.

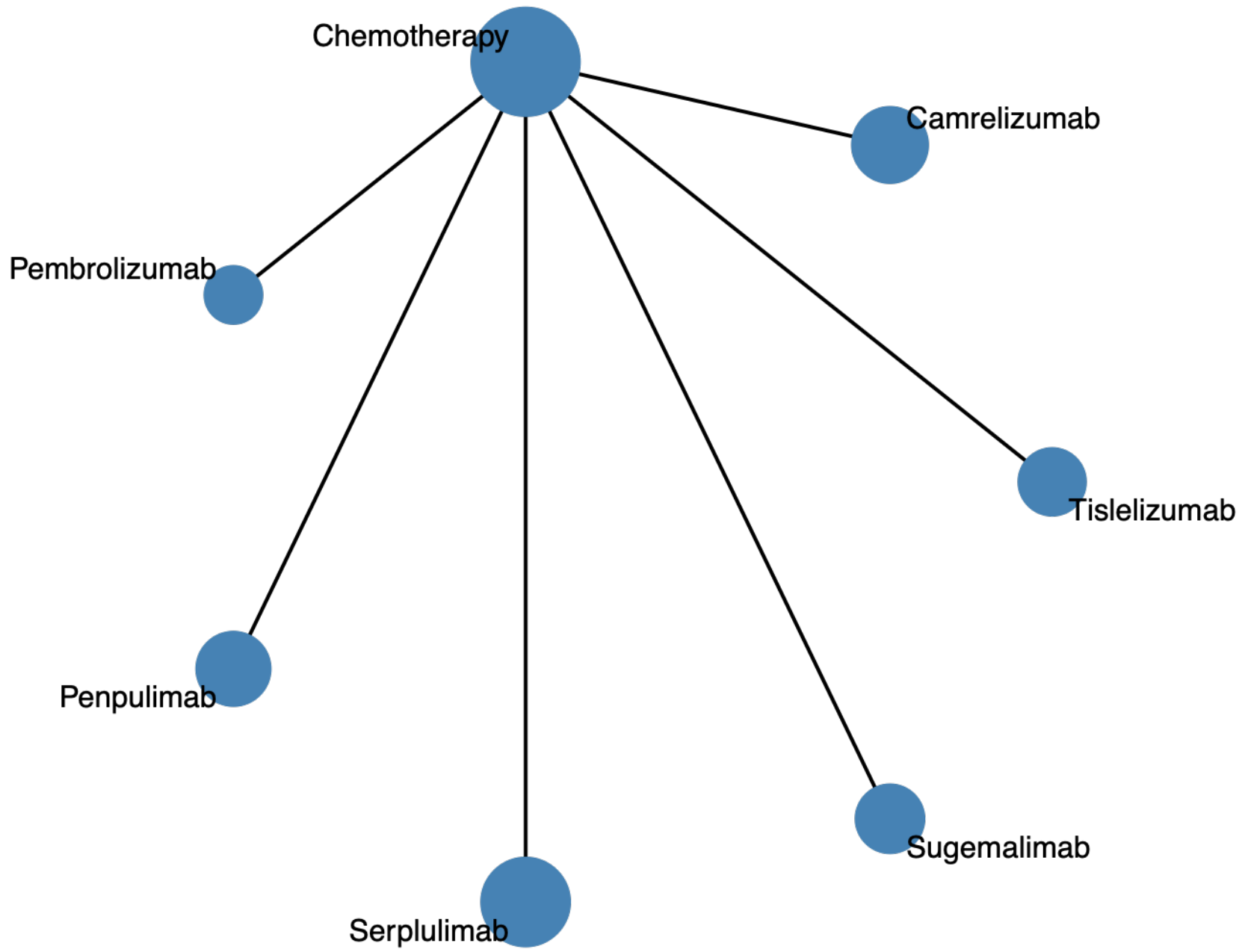

Figure S4. Forest plots of the treatment effects of six first-line ICI-plus-chemotherapy regimens versus chemotherapy alone. (**A**) Hazard ratios for overall survival. (**B**) Hazard ratios for progression-free survival.

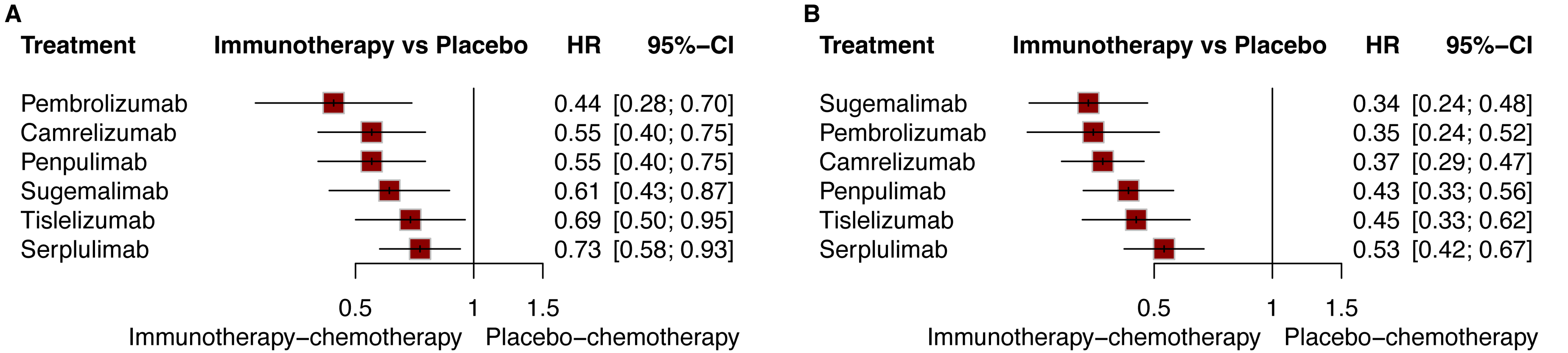

Figure S5. Cost-effectiveness acceptability curves for six ICI-based regimens and chemotherapy.

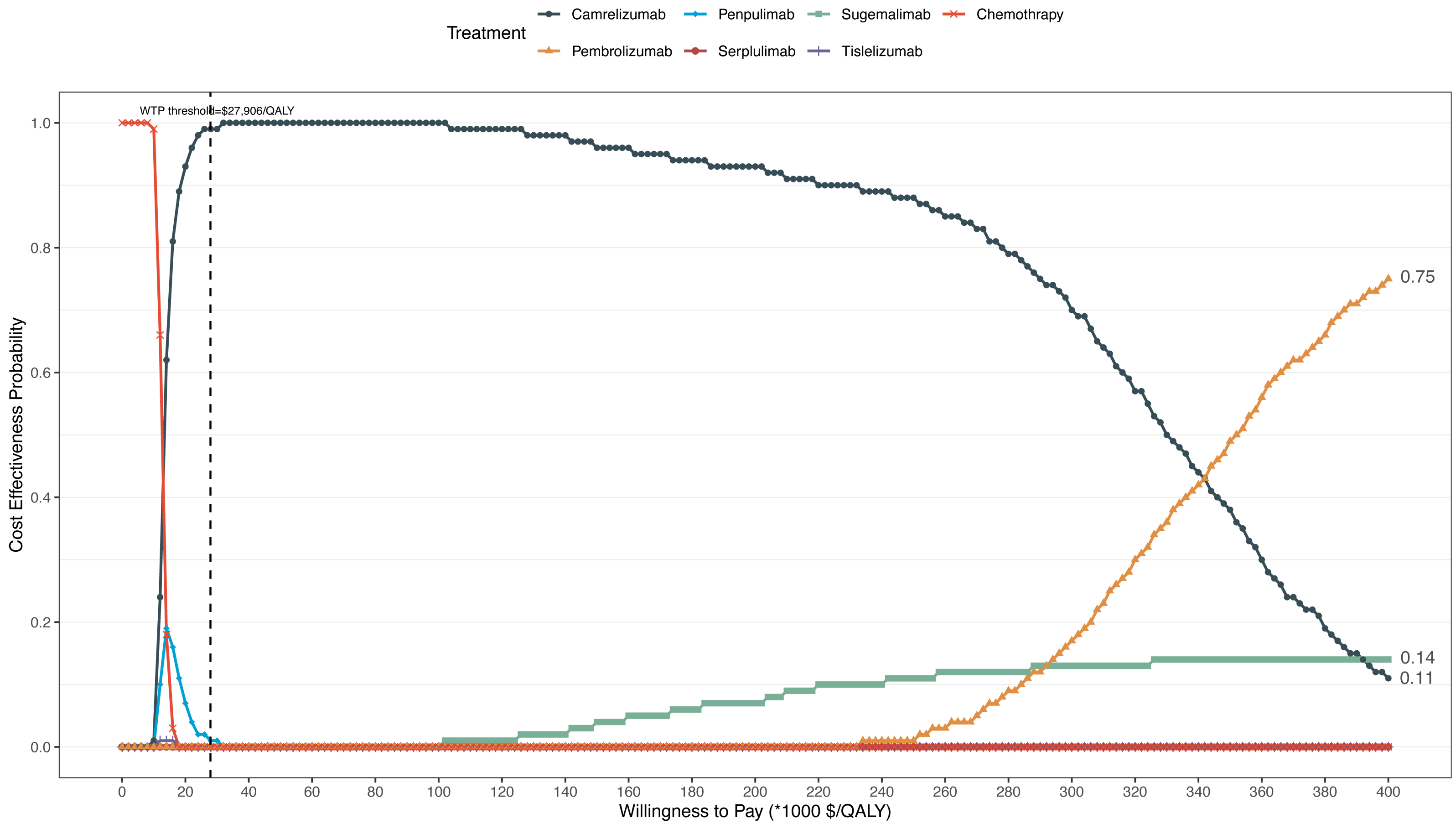

Abbreviation: WTP = willingness-to-pay; QALY = quality-adjusted life-year.

Figure S6. Probabilistic sensitivity analysis (**A**) and cost-effectiveness acceptability curves for penpulimab plus chemotherapy when the acquisition price of penpulimab was set at 100% (**B**), 90% (**C**), 75% (**D**), 50% (**E**), and 25% (**F**) of the current cost.

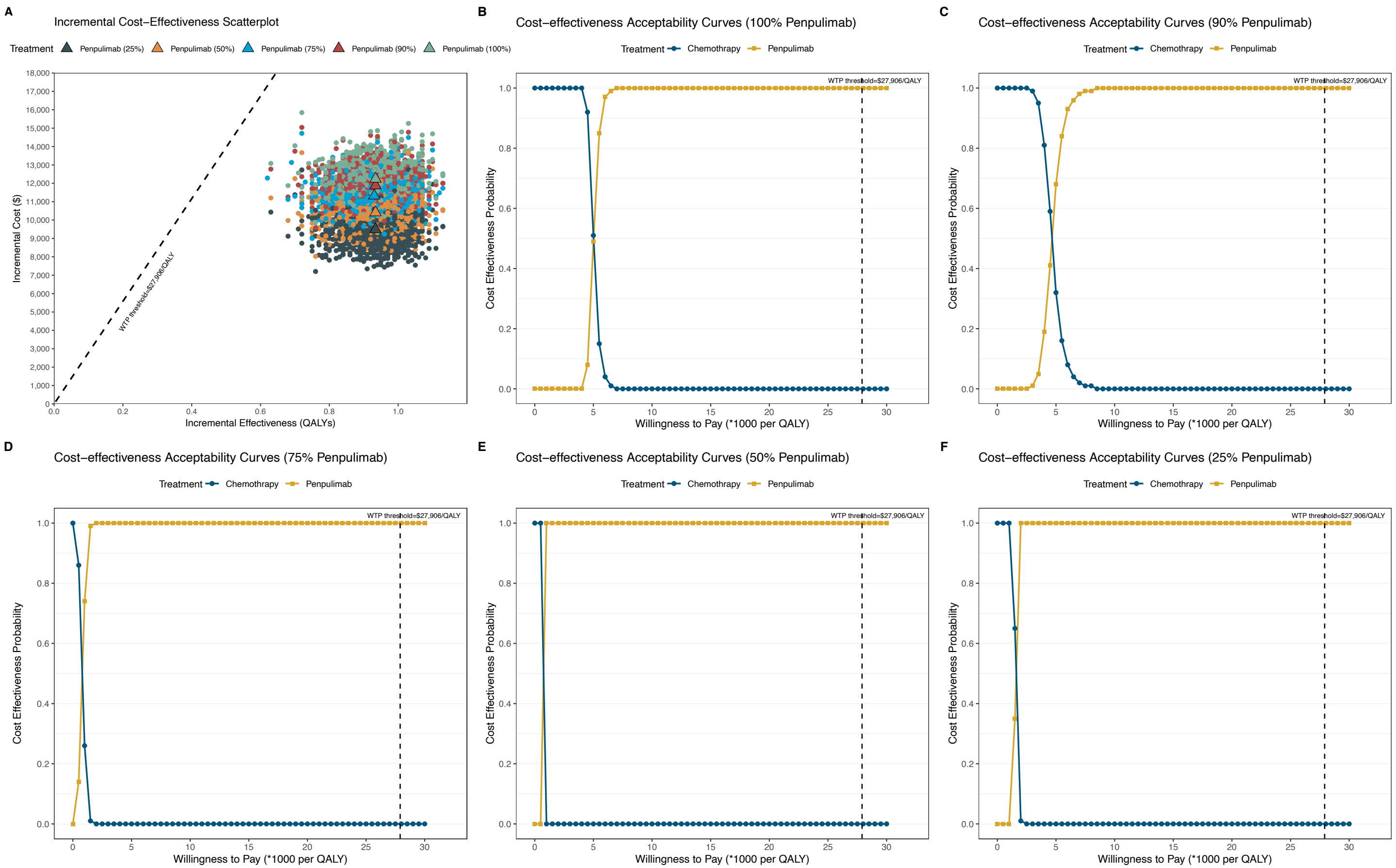

Abbreviation: WTP = willingness-to-pay; QALY = quality-adjusted life-year.

Figure S7. Probabilistic sensitivity analysis (**A**) and cost-effectiveness acceptability curves for tislelizumab plus chemotherapy when the acquisition price of tislelizumab was set at 100% (**B**), 90% (**C**), 75% (**D**), 50% (**E**), and 25% (**F**) of the current cost.

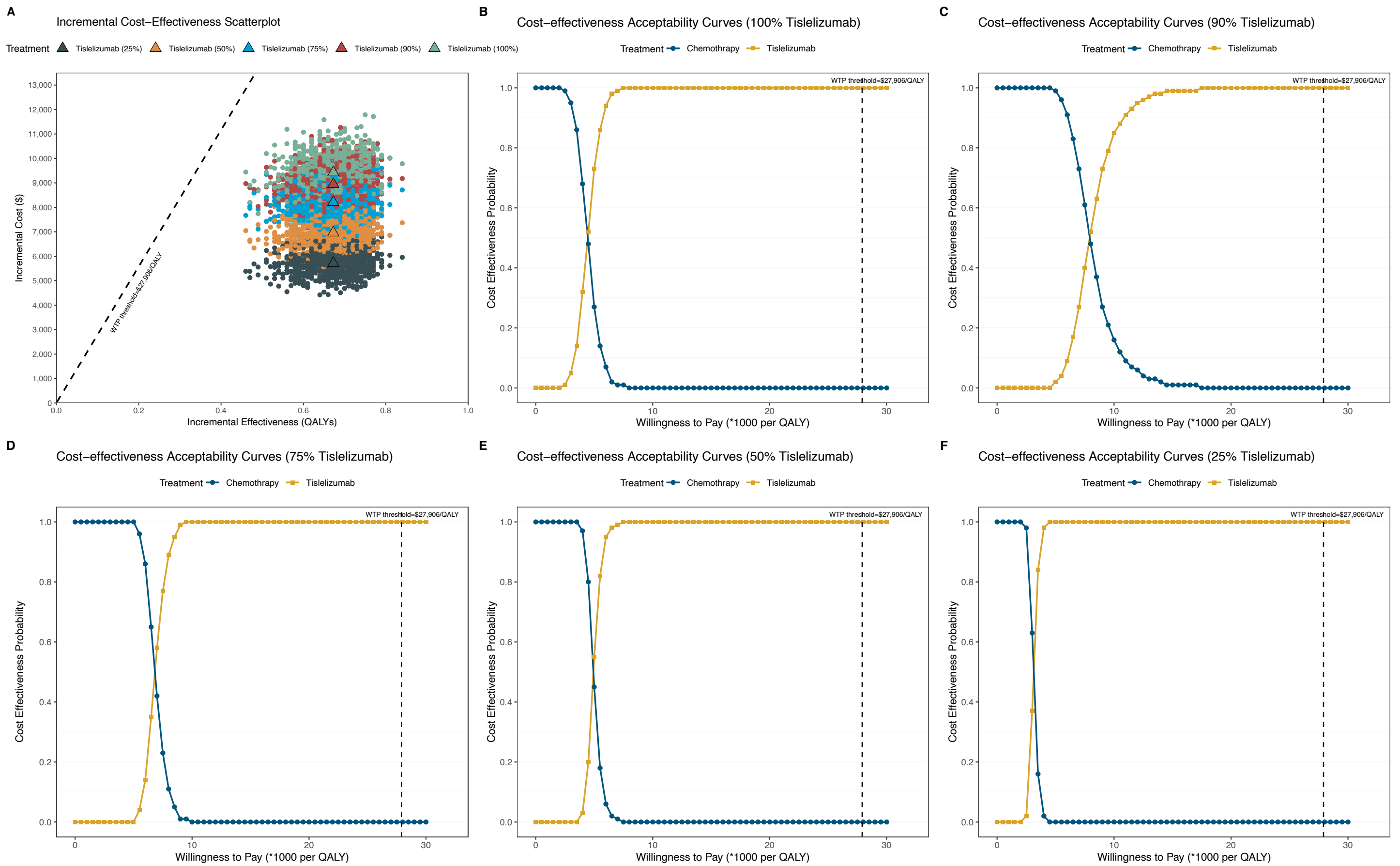

Abbreviation: WTP = willingness-to-pay; QALY = quality-adjusted life-year.

Figure S8. Probabilistic sensitivity analysis (**A**) and cost-effectiveness acceptability curves for sugemalimab plus chemotherapy when the acquisition price of sugemalimab was set at 100% (**B**), 90% (**C**), 75% (**D**), 50% (**E**), and 25% (**F**) of the current cost.

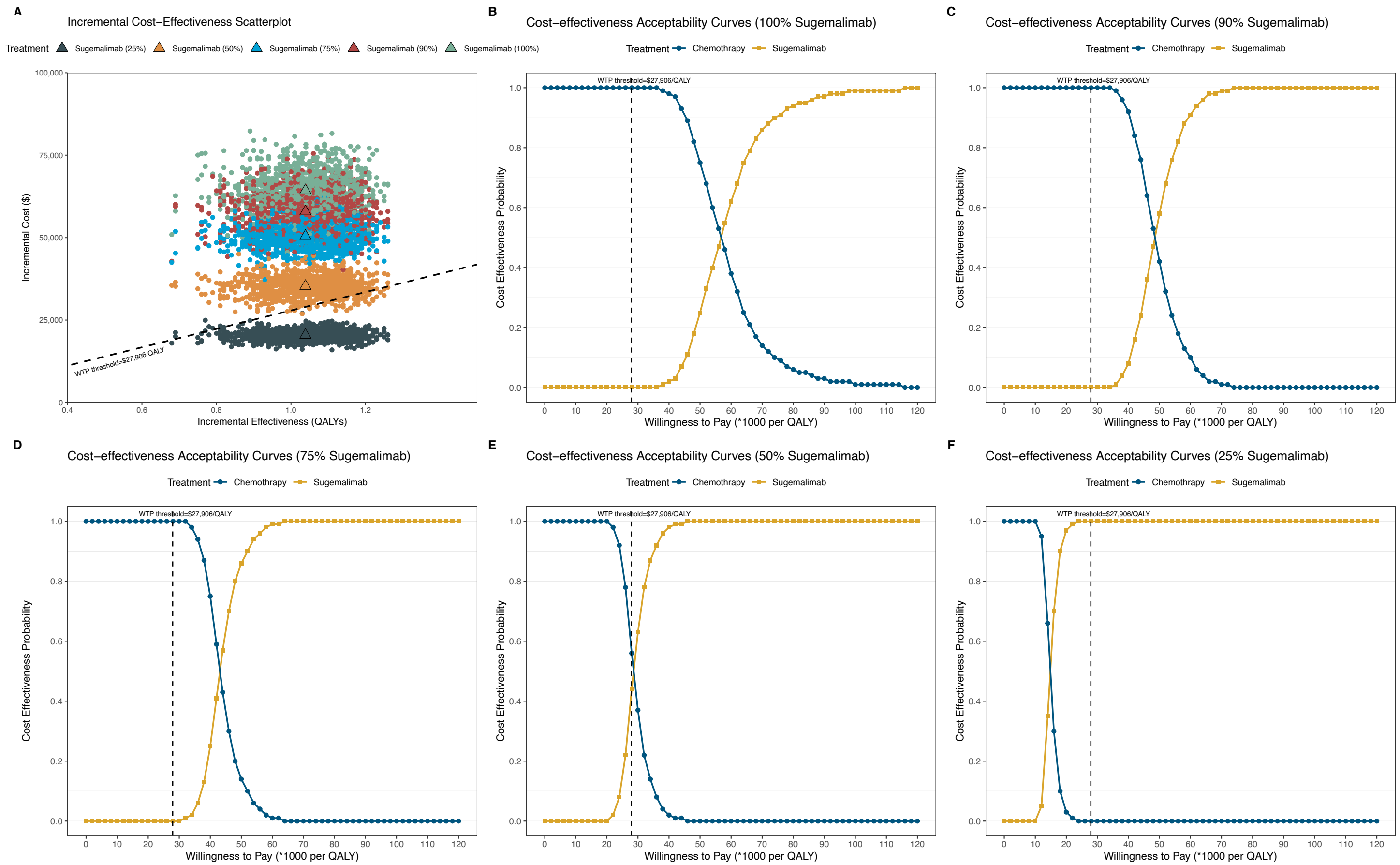

Abbreviation: WTP = willingness-to-pay; QALY = quality-adjusted life-year.

Figure S9. Probabilistic sensitivity analysis (**A**) and cost-effectiveness acceptability curves for serplulimab plus chemotherapy when the acquisition price of serplulimab was set at 100% (**B**), 90% (**C**), 75% (**D**), 50% (**E**), and 25% (**F**) of the current cost.

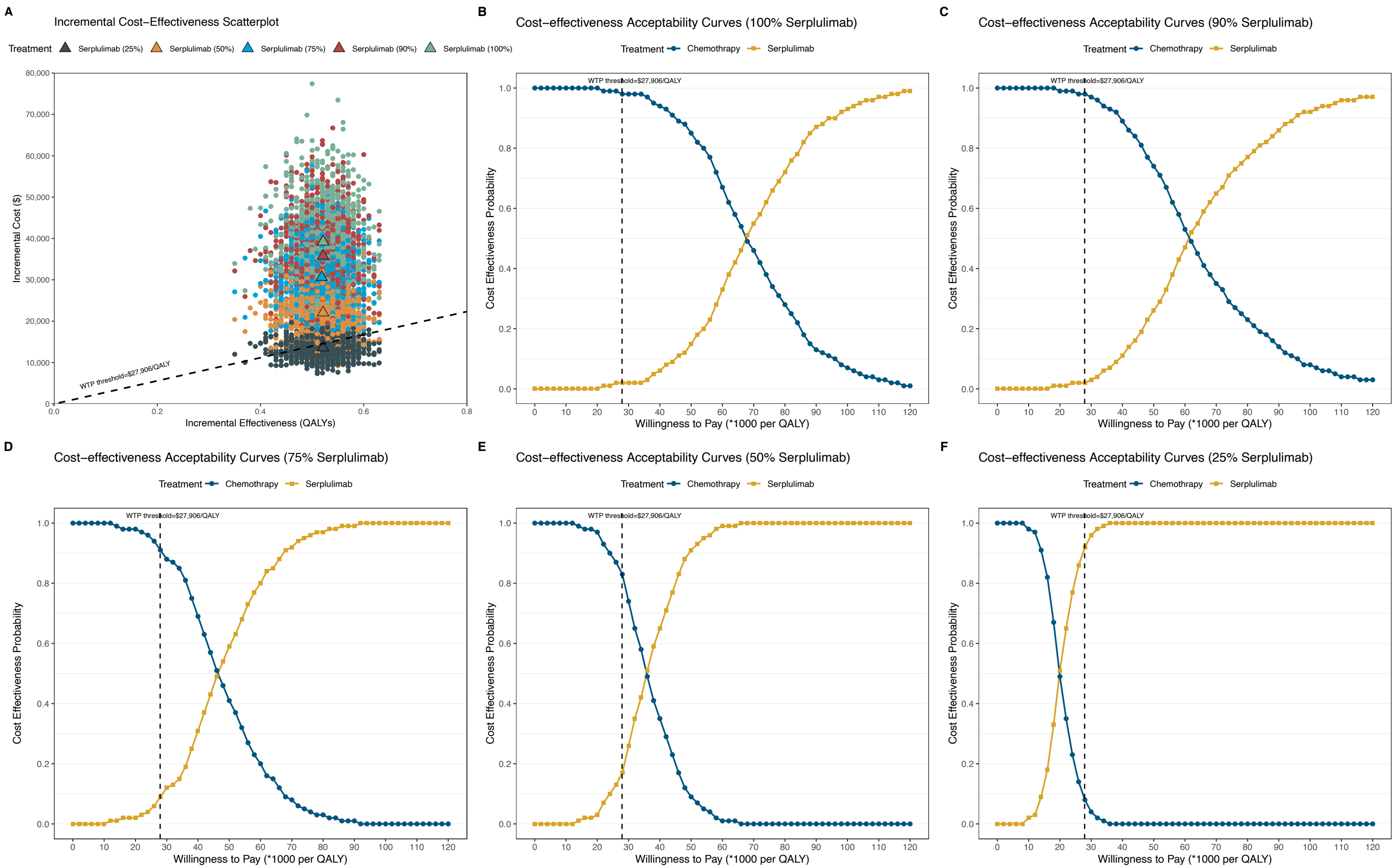

Abbreviation: WTP = willingness-to-pay; QALY = quality-adjusted life-year.
